# Malaria-Induced NLRP12/NLRP3-Dependent Caspase-1 Activation Mediates Inflammation and Hypersensitivity to Bacterial Superinfection

**DOI:** 10.1371/journal.ppat.1003885

**Published:** 2014-01-16

**Authors:** Marco A. Ataide, Warrison A. Andrade, Dario S. Zamboni, Donghai Wang, Maria do Carmo Souza, Bernardo S. Franklin, Samir Elian, Flaviano S. Martins, Dhelio Pereira, George Reed, Katherine A. Fitzgerald, Douglas T. Golenbock, Ricardo T. Gazzinelli

**Affiliations:** 1 Laboratório de Imunopatologia, Centro de Pesquisas René Rachou, Fundação Oswaldo Cruz, Belo Horizonte, Minas Gerais, Brazil; 2 Departamento de Bioquímica e Imunologia, Instituto de Ciências Biológicas, Universidade Federal de Minas Gerais, Belo Horizonte, Minas Gerais, Brazil; 3 Division of Infectious Diseases and Immunology, University of Massachusetts Medical School, Worcester, Massachusetts, United States of America; 4 Faculdade de Medicina de Ribeirão Preto, Universidade de São Paulo, Ribeirão Preto, São Paulo, Brazil; 5 Departamento de Microbiologia, Instituto de Ciências Biológicas, Universidade Federal de Minas Gerais, Belo Horizonte, Minas Gerais, Brazil; 6 Centro de Pesquisas em Medicina Tropical, Porto Velho, Rondônia, Brazil; Washington University School of Medicine, United States of America

## Abstract

Cyclic paroxysm and high fever are hallmarks of malaria and are associated with high levels of pyrogenic cytokines, including IL-1β. In this report, we describe a signature for the expression of inflammasome-related genes and caspase-1 activation in malaria. Indeed, when we infected mice, *Plasmodium* infection was sufficient to promote MyD88-mediated caspase-1 activation, dependent on IFN-γ-priming and the expression of inflammasome components ASC, P2X7R, NLRP3 and/or NLRP12. Pro-IL-1β expression required a second stimulation with LPS and was also dependent on IFN-γ-priming and functional TNFR1. As a consequence of *Plasmodium*-induced caspase-1 activation, mice produced extremely high levels of IL-1β upon a second microbial stimulus, and became hypersensitive to septic shock. Therapeutic intervention with IL-1 receptor antagonist prevented bacterial-induced lethality in rodents. Similar to mice, we observed a significantly increased frequency of circulating CD14^+^CD16^−^Caspase-1^+^ and CD14^dim^CD16^+^Caspase-1^+^ monocytes in peripheral blood mononuclear cells from febrile malaria patients. These cells readily produced large amounts of IL-1β after stimulation with LPS. Furthermore, we observed the presence of inflammasome complexes in monocytes from malaria patients containing either NLRP3 or NLRP12 pyroptosomes. We conclude that NLRP12/NLRP3-dependent activation of caspase-1 is likely to be a key event in mediating systemic production of IL-1β and hypersensitivity to secondary bacterial infection during malaria.

## Author Summary

Together *Plasmodium falciparum* and *P. vivax* infect approximately 250 million individuals, reaping life of near one million children every year. Extensive research on malaria pathogenesis has funneled into the consensus that the clinical manifestations are often a consequence of the systemic inflammation. Importantly, secondary bacterial and viral infections potentiate this inflammatory reaction being important co-factors for the development of severe disease. One of the hallmarks of malaria syndrome is the paroxysm, which is characterized by high fever associated with peak of parasitemia. In this study we dissected the mechanisms of induction and the importance of the pyrogenic cytokine, IL-1β in the pathogenesis of malaria. Our results demonstrate the critical role of the innate immune receptors named Toll-Like Receptors and inflammasome on induction, processing and release of active form of IL-1β during malaria. Importantly, we provide evidences that bacterial superinfection further potentiates the *Plasmodium-*induced systemic inflammation, leading to the release of bulk amounts of IL-1β and severe disease. Hence, this study uncovers new checkpoints that could be targeted for preventing systemic inflammation and severe malaria.

## Introduction

Every year, approximately 250 million people are infected with *Plasmodium*, contributing to significant social and economic instability in the developing countries around the world [1]. One of the main physiological responses to *Plasmodium* infection is the paroxysm – characterized by cycles of sharp peaks of high fever accompanied by chills and rigors, which coincide with the release of parasites from synchronized infected red blood cells [2], [3]. Parasite components, such as DNA bound to hemozoin [4], [5] and glycosylphosphatidylinositol (GPI) anchors [6], trigger the production of proinflammatory cytokines, including interleukin-1 beta (IL-1β), via activation of Toll-Like receptors (TLRs) [7]. Furthermore, malaria sepsis [8] leads to an exquisite sensitivity to secondary bacterial infections, in particular non-typhoidal salmonellosis, that often associate with severe disease [9]–[12]. Hence, a better understanding of the mechanisms involved on this inflammatory stage during malaria is critical for the clinical management and prevention of severe disease.

TLRs are only one family of the receptors required for the release of active IL-1β, as cleavage of pro-IL-1β by caspase-1 also requires activation of Nod-Like Receptors (NLRs) [13], [14]. Upon stimulation, the respective NLRs oligomerize and recruit pro-caspase-1 directly via a N-terminal caspase recruitment domain (CARD) homotypic interaction (CARD-CARD) (*e.g*., CARD-containing NLRs such as NLRP1 or NLRC4) or indirectly via the adaptor molecule called apoptosis-associated speck-like protein containing a caspase recruitment domain (ASC), as is the case of NLRP3-inflammasome [13]. The inflammasome assembly culminates on activation of caspase-1, and consequent release of the active form of IL-1β. NLRP3 containing inflammasome is activated in response to a large range of insults, such as pathogens, bacterial RNA, and crystal structures [15]–[17]. The NLRP12 was the first NLR shown to associate with ASC and to form an active IL-1β-maturing inflammasome [18]. This receptor was initially placed as a negative regulator of inflammation [19], [20], but it was also shown to be involved in periodic fever of cryopyrinopathies [21], and to mediate host resistance to *Yersinia pestis* [22].

Here, we asked what are the molecular steps required for and the physiological role of inflammasome assembly during malaria sepsis. Our results indicate that symptomatic *Plasmodium* infection triggers inflammasome formation and caspase-1 activation via an intricate process that requires several inflammatory mediators as well as NLRP3 and NLRP12. Furthermore, we found that the malaria-primed monocytic cells produce deleterious amounts of IL-1β when exposed to a second microbial challenge, being an important component of the overwhelming inflammatory response observed during bacterial superinfection.

## Results

### Expression of inflammasome genes and ASC-dependent caspase-1 activation in rodent malaria

**Figure 1:**
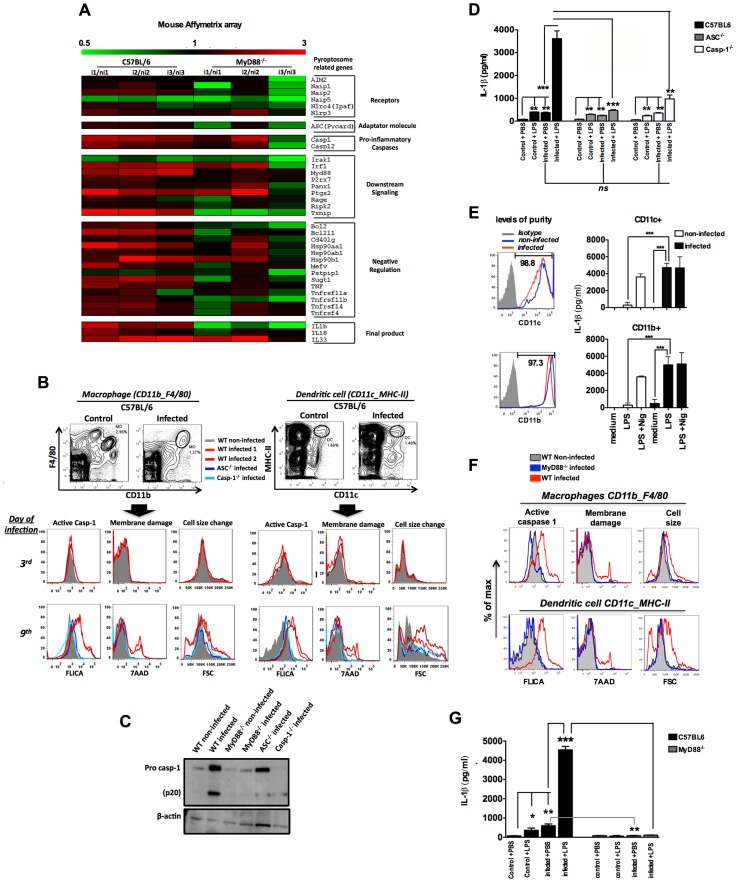
Caspase-1 activation, IL-1β production and pyroptosis in splenic macrophages and DCs from *P. chabaudi* infected mice. (**A**) Gene expression was determined in splenocytes of 3 C57BL/6 or MyD88^−/−^ mice at 6 days post-infection over 3 non-infected controls by Microarray analysis. (**B and F**) Splenocytes from C57BL/6, ASC^−/−^, Casp-1^−/−^ or MyD88^−/−^ mice were stained and analyzed by FACs to gate macrophages (CD11b^+^F4/80^+^) and DCs (CD11c^+^MHC-II^+^). Active caspase-1 was evaluated by FLICA reagent, membrane integrity by nuclei staining with 7AAD, and cell size change by shift on FSC axis. The results are representative from 3 experiments that yield similar results. (**C**) On day 7 post-infection splenocytes from C57BL/6, ASC^−/−^, Casp-1^−/−^ and MyD88^−/−^ mice were lysed by RIPA buffer and analyzed by Western blot employing an anti-caspase-1 antibody. A faint band of similar molecular weight of active caspase-1 corresponds to IgG light chain is seen in the infected ASC^−/−^ and Casp-1^−/−^ mice. (**D and G**) At 7 days post-infection mice were inoculated intravenously with 10 µg of LPS per mouse, and 9 hours later, sera was collected for measuring the levels of circulating IL-1β. The average levels of IL-1β in control and infected mice, before LPS challenge, were 64.2 and 434.2 pg/ml in figure D, and 82.2 and 602.4 pg/ml in figure G. These results are the means + SEM of 10–15 animals from 3 independent experiments that yield similar results. (**E**) CD11c^+^ and CD11b^+^ cells highly purified from spleens of C57BL/6 mice at day post-infection were cultured with LPS (1 µg/ml) and supernatants harvested 18 h later to measure the levels of IL-1β. As positive control we used the purified cells stimulated with LPS at same concentration followed by nigericin at 5 µM. Significant differences are indicated by *p<0.01, **p<0.001 and ***p<0.0005 obtained in the Mann-Whitney test.

The *Plasmodium chabaudi AS* rodent model was used to evaluate the *in vivo* activation of inflammasome. The microarray analysis of splenocytes from C57BL/6 at 6 days post-infection demonstrates enhanced expression of various genes from the inflammasome pathway, including *Casp1* and *Il1b* (**Figure 1A**). Consistently, the FLICA assay, which employs the fluorescent probe FAM-YVAD-FMK and Western blot, indicate that infection with *P. chabaudi* is sufficient to promote caspase-1 activation (**Figures S1A and** **1B**). Immunoblots evidenced enhanced expression and cleavage of pro-caspase-1 in spleens from *P. chabaudi* infected mice (**Figures S1B, S1C and** **1C**). The FLICA assay also revealed that macrophages (CD11b^+^F4/80^+^) and dendritic cells (DCs) (CD11c^+^MHC-II^+^) are the main source of active caspase-1 (**Figure 1B**) in the spleens from infected mice. We also observed a high frequency of macrophages and DCs undergoing inflammatory cell death (pyroptosis), as defined by damage of cell membranes evaluated by DNA-7AAD staining and augmented cell size associated with active caspase-1 (**Figure 1B**). Other splenic cell subsets did not express active caspase-1 or were undergoing pyroptosis during *P. chabaudi* infection (**Figure S1D**).

Importantly, macrophages and DCs from mice deficient for *Asc* (ASC^−/−^) and *Casp1* (Casp-1^−/−^) were negative for both active caspase-1 and pyroptosis markers during *P. chabaudi* infection (**Figures 1B**). Similar results were obtained when we used splenocyte lysates in immunoblots to detect active caspase-1 (**Figures 1C**). We also evaluated the role of caspase-1 activation in host resistance to *P. chabaudi*. C57BL/6, ASC^−/−^ and Casp-1^−/−^ mice injected with 10^5^ *P. chabaudi* infected erythrocytes displayed similar parasitemia beginning at day 5, peaking at days 7–8 days, and completely resolved at 25 days post-infection (**Figure S2A**). No lethality was observed until the end of the experiment.

Leuckocytes from mice and humans infected with *Plasmodium* are hyper-responsive to TLR agonists [23]. This hyperresponsiveness does not occur at all phases of infection. For example, as we show in Figure S2B, mice that are infected with *P. chabaudi* were hyperresponsive to small doses of TLR ligands, such as lipopolysaccharide (LPS), during the acute phase of the disease (day 7). This hyperresponsiveness was no longer observed when mice were challenged with low dose LPS 4 weeks after infection. Based on this and other studies [24], [25], we hypothesize that bacterial superinfection is a common co-factor for severe malaria. Thus, we used a challenge with low dose of bacterial LPS to mimic secondary bacterial infection and to evaluate the role of inflammasome and IL-1β in this process. C57BL/6 mice infected with *P. chabaudi* produced low, but significant levels of IL-1β, when compared to uninfected controls (**Figures 1D** **and S2B**). Surprisingly, these low levels of circulating IL-1β were produced in an ASC and Caspase-1-independent manner (**Figure 1D**). In contrast, production of IL-1β, which was extremely high in infected C57BL/6 mice challenged with LPS, was severely impaired in ASC^−/−^ or Casp-1^−/−^ mice (**Figure S2B and** **Figure 1D**). In order to check whether the active caspase-1^+^ cells were the main sources of active IL-1β, highly purified CD11b^+^ and CD11c^+^ cells from infected mice were stimulated with LPS and IL-1β measured thereafter. Both CD11b^+^ as well as CD11c^+^ cells produced high levels of IL-1β (**Figure 1E**).

### MyD88-dependent expression of inflammasome genes and caspase-1 activation in rodent malaria

The *in vivo* proinflammatory priming promoted by *Plasmodium* infection is partially dependent on TLR9 activation by parasite DNA [4], [23], [26]. Consistently, expression of various inflammasome genes was no longer enhanced in *Myd88-*deficient mice (MyD88^−/−^) infected with *P. chabaudi* (**Figure 1A** **and Table S1**). Accordingly, we did not observe active caspase-1, and pyroptotic features in macrophages and DCs from MyD88^−/−^ mice infected with *P. chabaudi* (**Figure 1C and 1F**). As expected, no systemic IL-1β was detected in sera of infected MyD88^−/−^ mice challenged or not with LPS (**Figure 1G**). Furthermore, the data presented in **Figure S3A** show reduced expression of pro-caspase-1 in *Tlr9*-deficient mice (TLR9^−/−^). In view of this reduced level of basal expression of the pro enzyme, it is not surprising that expression levels of its active form were decreased in TLR9^−/−^ mice infected with *P. chabaudi.* Likewise, the levels of circulating IL-1β were partially affected and survival rates increased in infected TLR9^−/−^ mice challenged with the low LPS dose (**Figures S3B and S3C**). Finally, we found that priming with CpG immunostimulatory oligonucleotides mimics *P. chabaudi* infection leading to enhanced susceptibility to LPS challenge, but does not induce lethality when used to challenge *P. chabaudi* infected mice (**Figure S3D**).

### IFN-γ is required for expression of pro-caspase-1 and pro-IL-1β in rodent malaria

**Figure 2:**
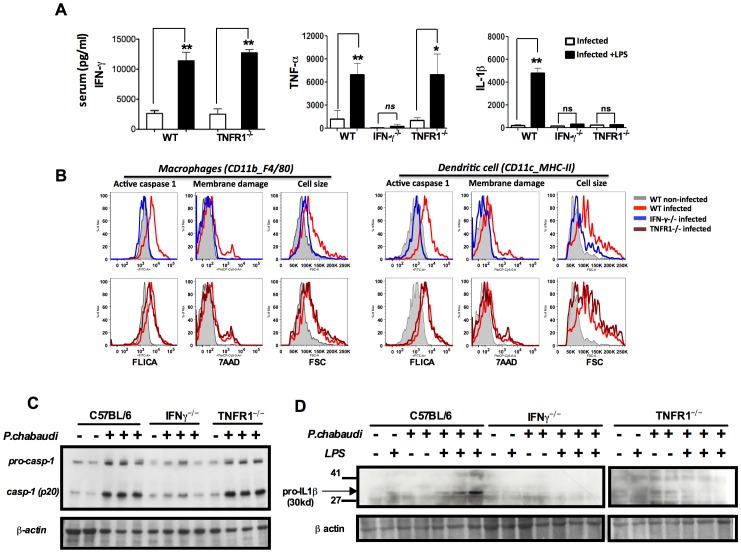
Both endogenous IFN-γ and TNF-α are required for IL-1β production in mice infected with *P. chabaudi*. (**A**) Mice were challenged with 10 µg of LPS, at 7 days post-infection, and sera collected 9 hours later for cytokine measurements. These results are means + SEM of 10 animals from 2 independent experiments. Significant differences are *p = 0.034 and **p = 0.001 as indicated by the Mann-Whitney test. (**B**) Active caspase-1, membrane integrity and cell size were assessed in macrophages (CD11b^+^F4/80^+^) and DCs (CD11c^+^MHC-II^+^) by flow cytometry, employing the FLICA reagent, nuclei staining with 7AAD, and the shift on FSC axis, respectively. These results presented in figures are representative of 2 experiments. (**C**) Splenocytes lysates were obtained from mice at 7 days post-infection and used in Western blot analysis. A faint band of similar molecular weight of active caspase-1 that corresponds to IgG light chain is seen in the uninfected controls or infected IFN-γ^−/−^ mice. These results presented in figures are representative of 2 experiments. (**D**) Two hours after LPS-challenge, splenocytes lysates were harvested to evaluate expression of pro-IL-1β. These results are representative of 3 independent experiment that yielded similar results.

The levels of circulating interferon gamma (IFN-γ), tumor necrosis factor-alpha (TNF-α) and IL-1β in sera of *P. chabaudi* infected mice, challenged or not with LPS, are presented in **Figure S2B**. Very low levels of IFN-γ, TNF-α, and IL-1β were detected in sera from uninfected mice challenged with low dose LPS. The hyperresponsiveness to LPS was observed in the first two weeks, but not at 28 days post-infection (**Figure S2B**). IFN-γ was shown to be a key mediator of inflammatory priming in febrile malaria patients and in mice infected with *P. chabaudi* [23]. Furthermore, TNF-α, a critical cytokine in malaria pathogenesis [27], [28] was shown to mediate expression of inflammasome components and pro-IL-1β [29], [30]. Indeed, even upon LPS challenge, active IL-1β was not produced in either *Ifng or Tnfrsf1a*-deficient mice (IFN-γ^−/−^) and (TNFR1^−/−^) respectively, when infected with *P. chabaudi* (**Figure 2A**). Normal activation of caspase-1 was observed in macrophages and DCs from TNFR1^−/−^, but not from IFN-γ^−/−^ mice (**Figures 2B, 2C** **and S4**). In contrast, both IFN-γ and TNF-α were required for expression of pro-IL-1β by spleen cells of infected mice challenged with LPS (**Figures 2D** **and S4**).

### NLRP3 and NLRP12 are required for caspase-1 activation and optimum IL-1β production during rodent malaria

**Figure 3:**
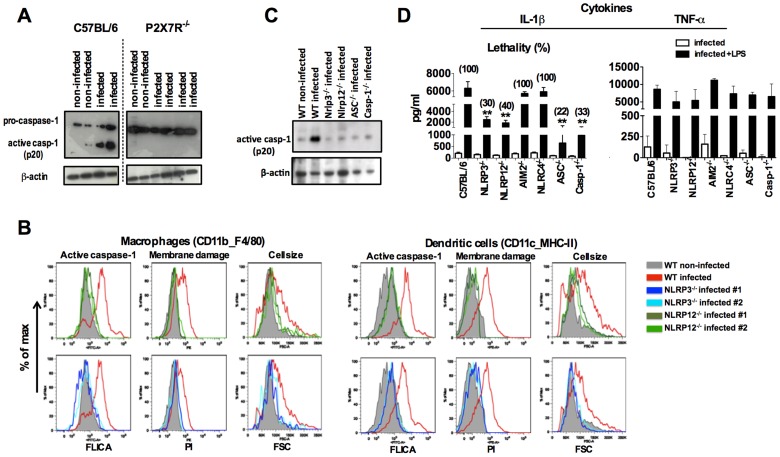
NLRP3/NLRP12-dependent activation of caspase-1 and pyroptosis in mice infected with *P. chabaudi*. (**A**) At 9 days post-infection, splenocytes from C57BL/6, and P2X7R^−/−^ mice were lysed and analyzed by Western blot employing caspase-1-specific antibody. (**B**) At 7 days post-infection, active caspase-1 by FLICA reagent, membrane integrity by propidium iodide, and cell size change by shift on FSC axis were assessed in splenic macrophages (CD11b^+^F4/80^+^) and DCs (CD11c^+^MHC-II^+^). (**C**) Splenocytes lysates from mice at 7 days post-infection were used to detected active caspase-1 by Western blot. A faint band of similar molecular weight of active caspase-1 that corresponds to IgG light chain is seen in the uninfected controls or in various infected knockout mice. The results presented in figures 3A, 3B and 3C are representative of 2 experiments that yield similar results. (**D**) A LPS dose of 10 µg/mouse was given intravenously at 7 days post-infection with *P.chabaudi* and sera collected 9 hours later, for measuring IL-1β and TNF-α levels. The numbers within parenthesis indicate the percentage of lethality 48 hours after LPS challenge (10 µg/mouse). The levels of IL-1β measured in the sera of infected C57BL/6, NLRP3^−/−^, NLRP12, ASC^−/−^ and Casp-1^−/−^ were not different. The results are means + SEM of 10 mice from 2 independent experiments. Significant differences are indicated by **p<0.001 obtained in the Mann-Whitney test.

We next evaluated the requirement of specific NLRs for caspase-1 activation. The P2X7 receptor (P2X7R) which senses extracellular ATP, opens a cation-specific channel that alters the ionic environment of the cell [31] culminating on NLRP3-inflammasome assembly under certain conditions [32], [33]. Here we found that P2X7R is necessary for caspase-1 activation during *in vivo* infection with *P. chabaudi* (**Figure 3A**). Consistently, NLRP3 and NLRP12 were required for activation of caspase-1, systemic production of IL-1β and pyroptosis (**Figures 3B, 3C and 3D**). Other cytosolic receptors, *i.e.* NLRC4 and absent in melanoma 2 (AIM2) were not essential for IL-1β release (**Figure 3D**). The levels of circulating IL-1β in sera of C57BL6, NLRP3^−/−^, NLRP12^−/−^, ASC^−/−^ as well as Casp-1^−/−^ infected mice, but no challenged with LPS, were not statically different (**Figure 3D**).

### Therapeutic effects of IL-1 receptor antagonist in rodent malaria

**Figure 4:**
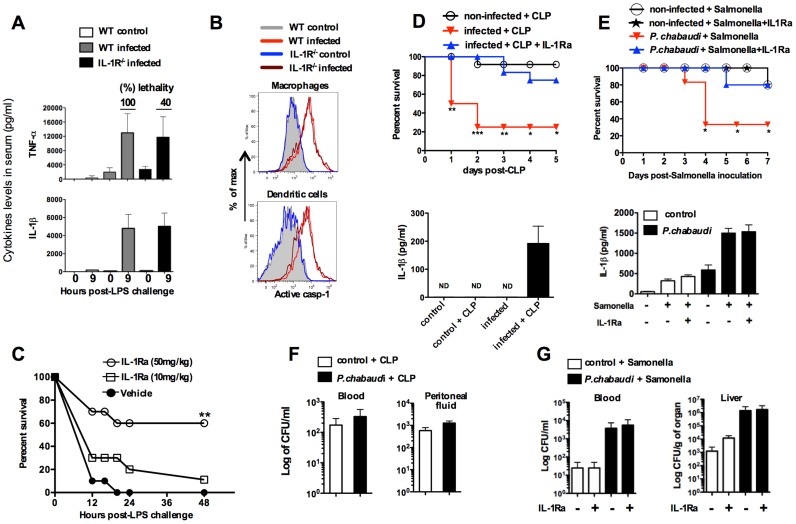
Treatment with IL-1RA prevents lethality in mice infected with *P. chabaudi* and challenged with a secondary bacterial infection. (**A**) At 7 days post-infection, mice were challenged with 10 µg of LPS and serum samples collected 9 hours later for cytokine measurements. The numbers within parenthesis indicate the percentage of lethality 24 hours after low dose (10 µg/mouse) LPS challenge. (**B**) Splenic macrophages (CD11b^+^F4/80^+^) and DCs (CD11c^+^MHC-II^+^) from mice at 7 days post-infection were stained with FLICA reagent in order to detect active caspase-1. (**C**) At day 7 post-infection the mice were treated with IL-1RA (anakinra) immediately prior to LPS challenge. Lethality was assessed from 12 to 48 hours post-LPS challenge. (**D**) At 7 days post-infection with *P. chabaudi*, sub-lethal sepsis was induced by CLP. A group of mice received treatment with IL-1RA (100 mg/kg/day) beginning 24 hours before the CLP procedure. Levels of circulating IL-1β were measured 24 hs after CLP. (**E**) Mice received peroral challenge with 10^8^ of *Salmonella typhimurium* at 5 days post-infection with *P. chabaudi.* A group of mice received treatment with IL-1RA (100 mg/kg/day) beginning 48 hours after bacterial challenge. The levels of circulating IL-1β were measured at 3 days post-*Salmonella* challenge. (**F**) Translocation of aerobic bacteria was quantified 24 hours after the CLP procedure. (**G**) Translocation of *S. typhimurium* was quantified 3 days after peroral challenge. We used 5 to 8 mice per group and results shown are representative of 2 independent experiments. Significant differences are *p<0.01, **p<0.005 ***p<0.001 obtained in a Chi-square test.

The infected *Il1r*-deficient mice (IL-1R^−/−^) mice are partially resistant to the LPS challenge, despite of the sustained levels of active caspase-1, IL-1β and TNF-α (**Figures 4A and 4B**). Importantly, in the different mouse lineages infected with *P. chabaudi*, we observed a striking correlation of high circulating IL-1β, but not necessarily active caspase-1, and lethality induced by LPS (**Table 1** **and** **Figure 3D**). Consistently, treatment with IL-1 receptor antagonist (IL-1RA) prevented lethality of *P. chabaudi* infected mice challenged with LPS (**Figure 4C**).

**Table 1:** Association of circulating IL-1β levels and high susceptibility to endotoxic shock in mice primed by infection with *P.chabaudi*.

Mice	Number of mice tested	Parasitemia(%)	Survival(%)	Active casp-1 MFI (×1000)	IL-1β levels (ng/ml)	Survival - Fischer's exact test wild type vs knockout mice
***C57BL/6 (wild type mice)***						
Uninfected control	15	-	100	1.57±0.17	0.35	
*P.chabaudi infected*	30	16	0	4.21±0.64	6.36	
***Mice deficient in TLR adaptor molecule and infected with P.chabaudi***						
MyD88^−/−^	*5*	*19*	*100*	*1.23*±*0.13*	*0.25*	*p<0.0001*
*Mice deficient in cytokine or in cytokine receptors and infected with P.chabaudi*						
IFN-γ ^−/−^	12	23	100	1.29±0.11	0.31	*p<0.0001*
TNFR1^−/−^	12	20	100	3.65±0.49	0.23	*p<0.0001*
IL-1R^−/−^	10	18.5	60	3.89±0.15	5.40	*p = 0.0012*
***Mice deficient in inflammasome components s and infected with P.chabaudi***						
ASC^−/−^	*8*	*17*	*78.5*	*1.12±0.23*	*0.47*	*p = 0.0003*
Casp-1^−/−^	*9*	*16*	*66.6*	*1.25±0.17*	*1.27*	*p = 0.0006*
NLRP3^−/−^	*10*	*17*	*70*	*1.18±0.27*	*2.61*	*p = 0.0002*
NLRP12^−/−^	*5*	*17.5*	*60*	*1.28±0.36*	*1.89*	*p = 0.0088*
AIM2^−/−^	*5*	*18*	*0*	*4.19±0.42*	*5.49*	*p = 1.00*
NLRC4^−/−^ (Ipaf^−/−^)	*4*	*14.5*	*0*	*3.93±0.32*	*4.38*	*p = 1.00*

IL-1β release and high susceptibility to endotoxic shock. Mice were infected with 10^5^ parasitized red blood cells (i.p). At 7 days post-infection, a low dose LPS (10 µg/mouse) was inoculated and lethality evaluated 24 hours later. Parasitemia was defined by smears giemsa- stained. FLICA reagent was used to assess active caspase-1 in total splenocytes from mice at 7 days post-infection. The data were collected by flow cytometry and median fluorescence intensity (MFI) analyses performed using Flowjo software. The range of detection of circulating IL-1β was 15.6–1000 pg/ml and determined using and ELISA Duoset kit from R&D Systems.

These results were validated in *P. chabaudi* infected mice challenged with sub-lethal cecal ligation puncture (CLP) (**Figure 4D**), a classic model for bacterial sepsis, as well as peroral infection with *Salmonella typhimurium* (**Figure 4E**). In both cases, bacterial superinfection leaded to rapid lethality that was associated with high circulating levels of IL-1β. Furthermore, mortality of co-infected mice was delayed or prevented by treatment with the IL-1RA. The loads of bacteria translocation were similar in co-infected mice treated or not with IL-1RA (**Figures 4F and 4G**).

### Increased frequency of pyroptotic CD14^dim^CD16^+^Caspase-1^+^ monocytes in PBMCs from symptomatic malaria patients

**Figure 5:**
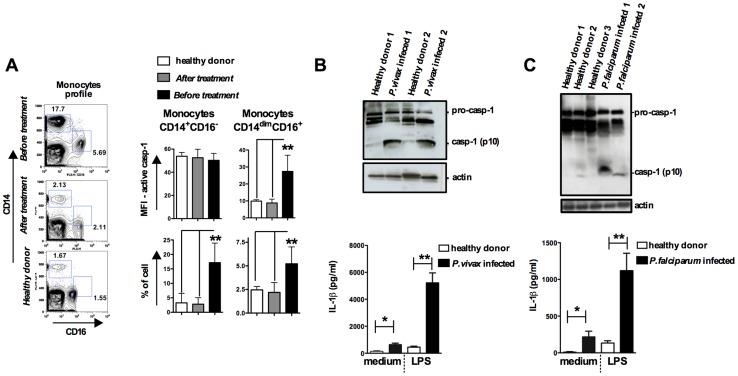
Monocytes are the major source of active caspase-1 during malaria. PBMCs were obtained from either *P. vivax* or *P. falciparum* malaria patients as well as from healthy donors. (**A**) The gate was set based on monocytes profile in PBMCs from *P. vivax* infected patients. PBMCs were stained with anti-CD14 and anti-CD16 antibodies to determine the presence of different monocyte subsets. These cells were gated based on FSC and SSC to avoid neutrophil contamination. When the CD14^dim^CD16^+^ gate was moved down in PBMCs from healthy donors or from malaria patients after treatment, we did not detect any active caspase-1. The bar graphs show the flow cytometry analysis of PBMCs from five *P. vivax* infected subjects before and after malaria treatment primaquine and chloroquine, as well as eight healthy donors. To determine the median fluorescence intensity (MFI) and frequency of (CD14^+^CD16^−^) and (CD14^dim^CD16^+^) that are active caspase-1, we used the FLICA assay. (**B – top panel**) Active caspase-1 (p10) was detected in lysates of PBMCs from *P. vivax* or (**C – top panel**) *P. falciparum* infected individuals by Western blot. (**B and C – bottom panel**) PBMCs were stimulated with LPS (100 ng/ml) for 24 hours, and levels of IL-1β assessed in the culture supernatants by ELISA. Significant differences are *p<*0.05* and **p<0.005 as indicated by the unpaired *t* test with Welch correction or Mann-Whitney test when a normality test failed.

We next studied caspase-1 activation and IL-1β release in peripheral blood mononuclear cells (PBMCs) from patients undergoing febrile malaria. Two different subsets of monocytes were closely examined: CD14^+^CD16^−^ or CD14^dim^CD16^+^ monocytes (**Figure 5A** **and S5A**). Active caspase-1 was constitutively expressed in CD14^+^CD16^−^ cells from healthy individuals, as previously described [34]. Nevertheless, the frequency of CD14^+^CD16^−^ cells was augmented on average 3 fold in *P. vivax* malaria patients. The frequency and intensity of caspase-1 expression, indicated by median fluorescence intensity (MFI), in different monocyte subsets from healthy and *P. vivax* infected individuals before and after treatment are shown in **Figure 5A**. Expression of active caspase-1 in association with membrane damage and augmented cell size was also observed in CD14^dim^CD16^+^ monocytes (**Figure S5A**). The representative histograms presented in **Figure S5A** were obtained from counter plot gates shown in **Figure 5A** and illustrate the results for active caspase-1, membrane damage and cell size change from one malaria patient before and after treatment, and a healthy donor. Other cell populations found in PBMCs were negative for active caspase-1 and pyroptosis markers (**Figure S5B**).

We also observed increased cleavage of caspase-1 (p10) in PBMCs from either *P. vivax* or *P. falciparum* malaria patients (**Figures 5B, 5C** **and S5C**). In addition, LPS-induced release of IL-1β is highly augmented in PBMCs from the same patients undergoing malaria sepsis (**Figure 5B and 5C** **– bottom panels**). Furthermore, we observed enhanced expression of inflammasome genes in PBMCs from *P. falciparum* malaria patients (**Figure S5D and Table S2**). As observed in the rodent malaria model (**Figure 2**), IFN-γ-priming of primary human monocytes mimics *in vivo* infection with *Plasmodium* and augments expression of pro-caspase-1, pro-IL-1β as well as IL-1β release induced by LPS stimulation (**Figure S5E**).

### NLRP12/NLRP3 containing inflammasomes in febrile *P. vivax* infected patients

**Figure 6:**
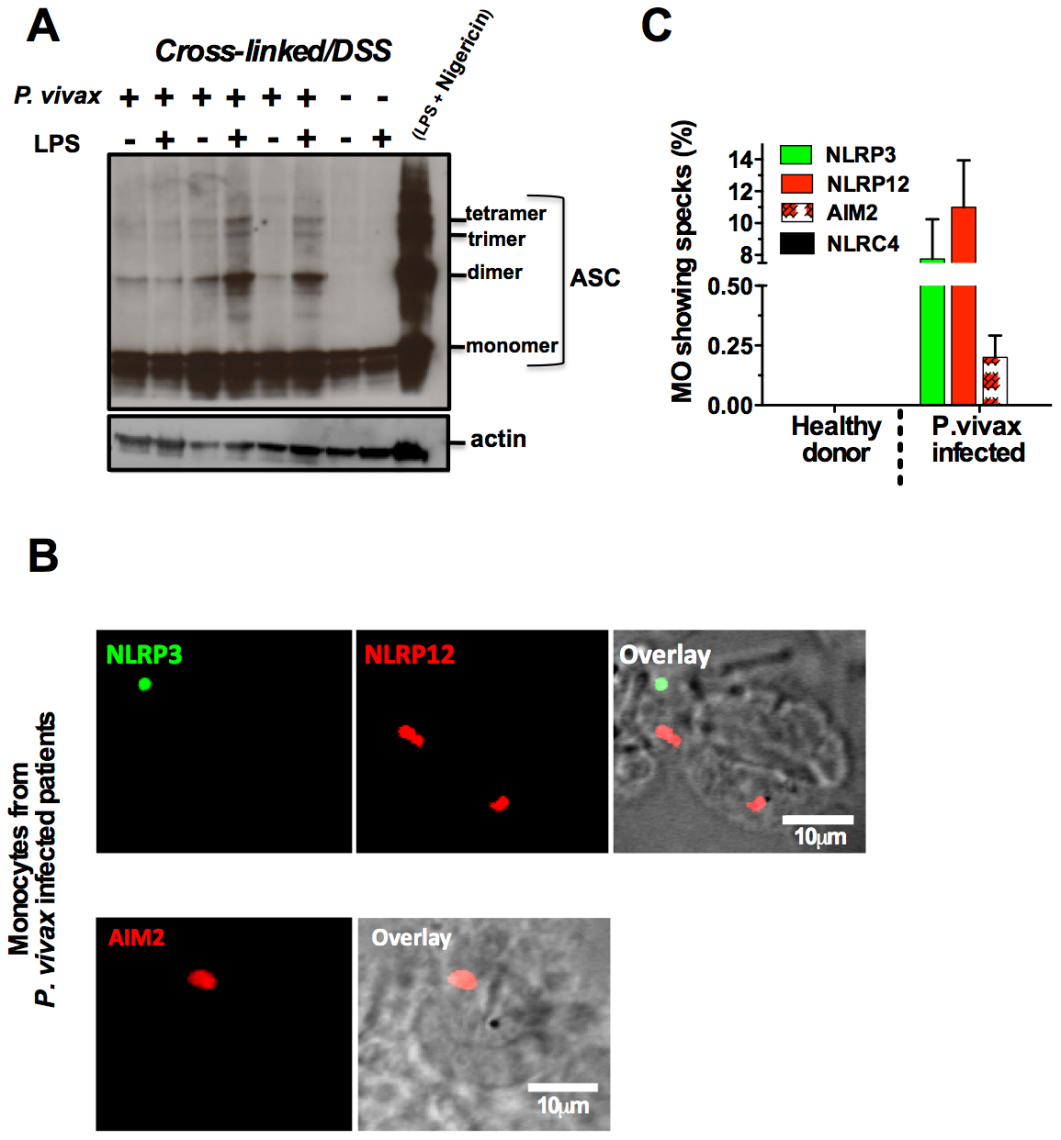
NLRP3/NLRP12 containing inflammasomes and caspase-1 activation in PBMCs from *P. vivax* malaria patients. (**A**) PBMCs derived from *P. vivax* malaria patients and healthy donors were lysed, cross-linked by treatment with disuccinimidyl suberate [70], and ASC oligomerization assessed by Western blot analysis. PBMCs from a healthy donor stimulated with LPS and nigericin were used as positive control. . (**B**) NLRP3, NLRP12 and AIM2 containing inflammasomes (specks) in monocytes from *P. vivax* malaria patients were visualized in a confocal microscope. (**C**) The bar graphs show the frequency of specks in monocytes derived from *P. vivax* malaria patients. We saw no specks on cells from healthy donors or cells from malaria patients incubated with the secondary antibody only. See also **Figure S6**.

The nature of malaria-induced inflammasome was further explored by performing a crosslinking assay and confocal analyses. We observed an augmented multimerization of ASC (**Figure 6A**) in PBMCs from *P. vivax* infected individuals. Additionally, confocal microscopy indicated that inflammasome specks contained either NLRP3 (green) or NLRP12 (red), were present in 7 and 10% of monocytes from *P. vivax* malaria patients, respectively (**Figure 6B and 6C**). There was no co-localization of NLRP3 and NLRP12 specks. We did not detect monocytes containing NLRC4-inflammasome, and AIM2 specks appeared in a low frequency, ∼0.25% of monocytes from infected individuals. No specks were detected in monocytes from uninfected healthy donors (**Figure 6C**). **Figure S6** provides controls of the confocal analysis.

## Discussion

Paroxysm, an acute fever accompanied by chills and rigors, is a hallmark of *Plasmodium* infection [3], [35]. While the physiological role of fever is controversial, it can aid in host defense, delaying the growth of pathogens with strict temperature preferences [36]. In fact, prior to the discovery of antibiotics, deliberate infection with *P. vivax* was used to induce high fever and eliminate infection with *Treponema pallidum* in neurosyphilis patients [37]. However, fever is also associated with various pathological processes, such as respiratory distress, anemia, and neurological manifestations that cause morbidity and mortality in malaria [3], [35]. These clinical manifestations are related to the intensity of the systemic inflammatory response, yet the basic details of malaria-induced cytokinemia are not understood. Overall, our results indicate that *Plasmodium* infection primes innate immune cells leading to the oligomerization of inflammasomes containing, ASC, NLRP3 and NLRP12, resulting in activation of caspase-1 and the production of copious amounts of IL-1β upon a second TLR activation. An immediate consequence of this inflammatory priming is a drastic reduction in the threshold to septic shock-like syndrome caused by secondary bacterial infection.

Although there is a controversy about the role of different TLRs in the pathogenesis of malaria [38]–[41], various studies indicate that the initial cytokine storm during malaria is driven by TLR activation. Initial studies suggested that GPI anchors were the main *P. falciparum* molecules responsible for eliciting the production of proinflammatory cytokines during malaria [6]. However, recent studies indicate that parasite derived DNA containing both immunostimulatory CpG and AT-rich motifs, is instead the main force driving the cytokine storm during malaria sepsis [4], [5], [42]. Indeed, mice bearing non-functional *TLR9* or *MyD88*, or treated with a TLR7/TLR9 antagonist display a less pronounced inflammatory response and attenuated pathology during experimental malaria [23], [26], [38], [41]. Moreover, mutations in *TLR2*, *TLR9* and *Mal/TIRAP* appear to affect the outcome of human disease [24], [25], [43], [44].

Importantly, infection with *Plasmodium* in humans leads to a proinflammatory priming and hyperresponsiveness to microbial products [23], [45]–[47]. This enhanced ability to respond to microbes during immunosurveillance protects the host against infectious insult, but the innate immune response is the classic “double-edged sword”. Thus, the proinflammatory priming means that the innate immune system can overreact to secondary infection, leading to a septic shock syndrome with clinical manifestations. It is noteworthy that malaria is often associated with bacterial infections [12]. Furthermore, a recent study highlights that the chance to develop severe malaria is elevated 8.5 fold in children with bacteremia [9]. Similarly, bacterial and viral infections may also act as co-factors for severe *P. vivax* malaria [10], [35], [48]. We propose that inflammasome and IL-1β [35], [49], [50] are important components of the proinflammatory priming and the exquisite sensitivity to superinfection during malaria.

Here, we demonstrate that both in mouse and human malaria expression of inflammasome genes, caspase-1 activation and pyroptosis are induced in phagocytic cells. As a consequence, during *Plasmodium* infection the threshold for LPS sensitivity is decreased in at least 100 folds, as compared to non-infected control mice [51], [52]. Previous studies have shown that 1.0 mg of LPS induces the production of high IL-1β levels and lethality [52], [53]. In our model, challenge with 10 µg of LPS did not promoted or augmented caspase-1 activation in either uninfected or infected mice, respectively. Hence, *Plasmodium* infection is sufficient to induce inflammasome assembly and caspase-1 activation, but requires a challenge with a low dose of LPS to induce expression of pro-IL-1β and release of high levels of its active form.

Our studies in the *P. chabaudi* mouse model demonstrate that expression of inflammasome genes; pro-caspase-1 as well as pro-IL-1β are all dependent on intact *Myd88* function. The MyD88 role on the process is independent of the IL-1 receptor signaling, as macrophages and DCs from *P. chabaudi-*infected IL-1R^−/−^ mice show normal levels of caspase-1 activation and pyroptosis. In fact, we found that TLR9 explains, in part, the role of MyD88 on caspase-1 activation. As previously reported [54], treatment with CpG oligonucleotides mimics the *P. chabaudi* infection making the mice more susceptible to septic shock. Curiously, challenge of infected mice with CpG oligonucleotides did not result in lethality. As CpG DNA and LPS preferentially target DCs and monocyte/macrophages, respectively [55], our data suggest that the nature of the ligand and differential expression of TLRs are important determinants on priming and cytokine production by *Plasmodium* infected mice challenged with LPS.

We hypothesize that by targeting TLR9 on DCs, *Plasmodium* infection elicits the IL-12 production, and consequent IFN-γ-dependent inflammatory priming [23]. Priming with IFN-γ mediates caspase-1 activation through the induction of pro-caspase-1 expression, and the lack of either IFN-γ or TNFR1 results in impaired expression of pro- IL-1β. Hence, LPS, but not CpG, stimulates the production of high levels of TNF-α and pro-IL-1β culminating on the release of deleterious amounts of active IL-1β by IFN-γ-primed monocytes/macrophages. An alternative explanation is that by inducing Type I IFN production by DCs, *Plasmodium* infection inhibits the excessive production of IL-1β [56], [57], and prevents lethality induced by TLR9 activation.

**Figure 7:**
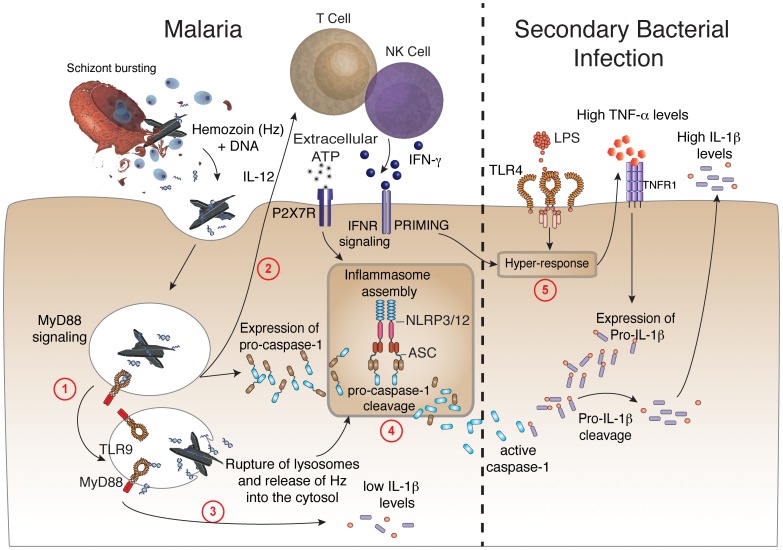
Malaria-induced NLRP12/NLRP3-dependent caspase-1 activation mediates IL-1β and hypersensitivity to bacterial superinfection. Step 1 –Phagocytes internalize *Plasmodium* DNA bound to hemozoin that activates TLR9 and the adaptor molecule named MyD88. **Step 2 –** MyD88 signaling triggers the expression of IL-12, which will initiate the production of IFN-γ by T lymphocytes and NK cells. **Step 3 –** Low levels of caspase-1-independent IL-1β induced by malaria infection. **Step 4 –** IFN-γ priming and MyD88 signaling in phagocytes will lead to enhanced expression of pro-caspase-1. K^+^ efflux as well as rupture (by hemozoin crystals) and release of lysosome contents will induce the assembly of ASC, NLRP3 and NLR12 inflammasomes and promote cleavage of pro-caspase-1. **Step 5 -** Bacterial superinfection triggers expression of high pro-IL-1β levels, in a TNF-α-dependent manner. Pro-IL-1β will be cleaved by active caspase-1 generated on **step 4**. Upon secondary bacterial infection, the malaria-primed macrophages will release deleterious amounts of IL-1β.

Another important requirement for caspase-1 activation in rodent malaria was the purinergic P2X7 receptor that under certain conditions mediates NLRP3-inflammasome activation [32], [58]. Indeed, *in vitro* experiments reported that synthetic hemozoin induces inflammasome formation, activation of caspase-1 and release of IL-1β by macrophages via NLRP3 [59]–[62]. Here, we demonstrated an *in vivo* requirement of both NLRP3 and NLRP12 for inflammasome formation and caspase-1 activation during *in vivo* infection with *P. chabaudi.* We also report that symptomatic infection with either *P. falciparum* or *P. vivax* leads to enhanced expression of inflammasome related genes and caspase-1 activation. Notably, we notice an *in vivo* assembly of NLRP3 and NLRP12 specks and ASC oligomerization in febrile malaria patients. A diagram detailing the different steps required for inflammasome assembly during malaria sepsis, and release of copious amounts of IL-1β during bacterial superinfection is presented in **Figure 7**.

It is noteworthy that periodic fever is a main symptom of cryopyrinopathies, a human inflammatory disorder that is associated with mutations in both *NLRP3* and *NLRP12* genes [21]. These patients have less severe symptoms when treated with IL-1R antagonist [63]. However, the relevance of this process during *in vivo Plasmodium* infection is controversial. While Dostert and colleagues [59] demonstrated a partial protection to experimental cerebral malaria in NLRP3^−/−^ mice, other studies reported that this pathological process occurs independent of NLRP3, ASC, Caspase-1, IL-1R, IL-1β, and IL-18 [62], [64]. Furthermore, a study in the *P. chabaudi* model shows that caspase-12 (but not caspase-1), modulates cytokine responses and development of acquired immunity [65]. Thus, our results indicate that bacterial superinfection overcome the regulatory role of caspase 12.

Our results demonstrate that *P. chabaudi* infection triggers low levels of IL-1β release in an inflammasome-independent manner. Nevertheless, the parasite-induced NLRP3 and NLRP12 inflammasomes play a key role in the release of high IL-1β levels and hypersensitivity to LPS during malaria sepsis. Importantly, the lethality induced by low dose LPS, peroral infection with *S. typhimurium*, or sublethal CLP in *P. chabaudi* infected mice was prevented or delayed by treatment with IL-1R antagonist.

Relevant to our findings is the recently published study demonstrating that malaria impairs host resistance to *Salmonella* infection [11]. They propose that induction of heme oxygenase −1 (HO-1) by *Plasmodium* infection limits the generation of reactive oxygen species (ROS), an important mechanism of host resistance to *Salmonella* infection [66]. Another consequence of the decreased ROS generation during malaria would be the uncontrolled activation of caspase-1 and release copious amounts of active IL-1β by phagocytes, as previously reported in patients with chronic granulomatous disease (CGD) [67]–[69].

Similarly to murine malaria, extremely high levels of IL-1β are produced by PBMCs from *P. vivax* or *P. falciparum* malaria patients exposed to bacterial components [23], [47]. Importantly, the bacterial load in mice undergoing malaria sepsis was not different from mice treated with IL-1RA, suggesting that the acute lethality caused by bacterial superinfection is due to the deleterious inflammatory response. Overall, our data argue that the IFN-γ, TNF-α and MyD88 role on hypersensitivity to septic shock during malaria is, at least in part, mediated by inflammasome-dependent release of IL-1β.

In conclusion, years of research on malaria pathogenesis have funneled into the consensus that the clinical manifestations are often a result of the excessive activation of the innate immune cells. Recent reports have emphasized the important role of bacterial infections as co-factors for severe disease. Here we report that *Plasmodium*-induced NLRP3, NLRP12, ASC containing inflammasomes and caspase-1 activation, which are important events for the overwhelming IL-1β response and morbidity observed in bacterial superinfection during malaria sepsis.

## Materials and Methods

### Ethics statement

The study with *Plasmodium* infected patients and healthy controls was approved by the Ethical Committee of Research (CEP) from the Research Center of Tropical Medicine (CEP-CEPEM 096/09); the Brazilian National Committee of Research (CONEP/Ministry of Health – 15653); as well as the Institutional Research Board from the University of Massachusetts Medical School (UMMS) (IRB-ID11116_1). Informed written consent was obtained before enrollment of all subjects (*Plasmodium* infected patients and healthy control). All experiments involving animals were in accordance with guidelines set forth by the American Association for Laboratory Animal Science (AALAS) and with the recommendations in the Guide for the Care and Use of Laboratory Animals of the Brazilian National Council of Animal Experimentation (http://www.cobea.org.br/) and the Federal Law 11.794 (October 8, 2008). All protocols developed for this work were approved by the Institutional Animal Care and Use Committee (IACUC) at the UMMS (ID - 2371-12), (ID - 1369-11) and also were approved by the Council of Animal Experimentation of Oswaldo Cruz Foundation (CEUA protocol 38/10-3).

### Reagents

LPS O55:B55 from *E. coli*, nigericin and RIPA buffer were obtained from SIGMA. All mAbs used in flow cytometry, as well as 7AAD and PI were purchased from Ebiosciences. Active caspase-1 detection kit was obtained from ImmunoChemistry Technologies, LLC (catalog no. 98). Human (SC-515) and mouse (SC-514) anti-caspase-1 (p10), ASC (SC-22514-R and SC-271054) primary antibodies were obtained from Santa Cruz Biotechnology and anti-actin (A2066) was purchased from SIGMA. Anti-caspase-1 (p20) was donated by Genentech. Anti-NLRP3 (ab4207 and ab17267), -NLRP12 (ab64928 and ab57906), -NLRC4 (ab99860), secondary Anti-rabbit IgG Texas Red (ab6800), secondary anti-goat IgG Texas Red (ab6883), secondary anti-goat IgG FITC (ab6881), and secondary anti-mouse IgG FITC (ab7057) were purchased from Abcam. The secondary antibodies used in western blots were purchased from KPL. Ficoll-Paque from GE Healthcare, and the crosslinker disuccinimidyl suberate [70] was obtained from Thermo Scientific. Protease inhibitor (EDTA free) was purchased from Roche and RPMI and DMEM from Gibco. Cytokine ELISA kits and Cytometric Bead Array were obtained from R&D Systems and BD Biosciences, respectively. Flow cytometry mAbs for mouse were from BD Biosciences: CD11c-PE (cat-557401), CD11b-PE (cat-557397), CD3-FITC (cat-553062), B220-APC (cat-561880), and from Ebioscience: MHC-II-PercpCy5 (cat-15-5321-82), and F4/80-APC (cat-17-4801-82), CD4-PE (cat-12-0041), CD8-PE (cat-12-008-81), and NKG2d-PE (cat-12-5872). Flow cytometry mAbs for human were from BD Biosciences: CD16-FITC (cat-555969), CD14-APC (cat-561708), CD19-FITC (cat-555412); and from Ebioscience: CD3-FITC (cat-55332), CD4-PE-Cy5 (cat-555348), CD8-PE-Cy5 (cat-555368), CD1c-APC (cat-17-0015), CD123-PercpCy5.5 (cat-45-1239), CD303-FITC (cat-11-9818-42), CD56-PE (cat-9012-0567).

### Malaria patients

Patients with acute febrile malaria were seen in the outpatient malaria clinic in the Tropical Medicine Research Center in Porto Velho, Brazil. Patients infected with *P. falciparum* received a fixed dose of the artemeter (20 mg) and lumefetrine (120 mg) combination four times a day for three days. Patients infected with *P. vivax* were treated with chloroquine (150 mg) every 8 hours for three days and primaquine (15 mg) in a single dose per day for two weeks. Up to 100 cc of blood was obtained immediately after confirmation and differentiation of *Plasmodium* infection by a standard peripheral smear; and 30–40 days after therapy with confirmed parasitological cure by PCR. Non-infected subjects living in Porto Velho were also included in the study.

### Mice and parasite

The knockout mice, ASC**^−/−^**, NLRP3**^−/−^**, and NLRP12**^−/−^** mice were generated by Millennium Pharmaceuticals and backcrossed 8–11 generations to C57BL/6 background. MyD88**^−/−^** and NLRC4^−/−^ mice were provided by S. Akira and R. Flavel. AIM2^−/−^ mice were provided by K. Fitzgerald. C57BL/6, IL-1R**^−/−^**, TNFR1**^−/−^**, P2X7R**^−/−^** and IFN-γ^−/−^ mice were purchased from Jackson Laboratories. The caspase-1 knockout mice used in this work was originally provided by Dr. Flavell from Yale University School of Medicine. All mice used in experiments were 8–12 weeks of age and bred in isolated conditions in the animal house at CPqRR or at UMMS Animal Facility.

The *Plasmodium chabaudi chabaudi AS* strain was used for experimental infections. This strain was kept in our laboratory as described elsewhere [71]. Briefly, *P. chabaudi* strain was maintained in C57BL/6 mice by passages once a week. For experimental infection mice were injected i.p. with 10^5^ infected red blood cells and parasitemia followed every three days. Although animals exhibit signs of disease, lethal infection is uncommon. The course of parasitemia in WT mice was similar to that reported in other studies [23], [72], .

### Cecal ligation puncture (CLP) and *S. typhimurium* infection

For sub-lethal sepsis induced by CLP, mice were anesthetized, incision made on the anterior abdomen, cecal ligated and punctured two times with a 22-gauge needle. Bacterial load in exudates from the peritoneal cavity and blood 24 hours after CLP was evaluated on Mueller-Hinton agar dishes [74]. For *Salmonella* infections mice were inoculated intragastrically with *Salmonella enterica* serovar Typhimurium (ATCC 14028) (10^8^ cfu). Three days after infection, mice were euthanized, liver aseptically collected, weighed, and homogenized in sterile PBS (1∶10, w/v). One hundred µl aliquots of serial decimal dilutions of liver homogenates and blood were plated onto MacConkey agar [75].

### PBMC and monocytes

PBMCs were isolated from whole blood on Ficoll-paque Plus (GE Healthcare). Cells were then plated into 96-well cell culture plates at a density of 2×10^5^ in DMEM containing 10% FCS and 10 µg/ml ciprofloxacin. Supernatants were collected 24 hours after stimulation and used to determine the levels IL-1β. Monocytes were purified from PBMCs of *P. vivax* infected patients and healthy donors by using a kit based on immunomagnetic negative selection from StemCell Technologies (catalog number 19058).

### FACS analysis for caspase-1 activation, membrane damage, and change in cell size

PBMCs from acutely infected patients were stained with combinations of the following mAbs: monocytes (CD14/CD16), T lymphocytes (CD3^+^/CD4^+^ or CD3^+^/CD8^+^), B lymphocytes (CD19), myeloid DCs (CD1c^+^/CD19^−^), plasmacytoid DCs (CD123^+^/CD303^+^) and NK cells (CD3^−^/CD56^+^). Splenocytes from infected mice were stained with combinations of the following mAbs: macrophages (CD11b^+^/F4/80^+^), DCs (CD11c^+^/MHC-II^+^), T lymphocytes (CD3^+^/CD4^+^ or CD3^+^/CD8^+^), B lymphocytes (B220^+^) and NK cells (NKG2d^+^). To each sample, FLICA reagent and PI (or 7AAD) were added as indicated. The data were acquired using a LSRII cytometer.

### Immunoblots for caspase-1 and pro-IL-1β

Ripa buffer (250 µl) plus protease inhibitor cocktail from Roche were added to a pellet containing 4×10^7^ splenocytes or PBMCs. After 15 minutes on ice, lysates were centrifuged at 13,000 *g* for 20 min at 4°C. The supernatants were separated in a 15%-acrylamide SDS-PAGE, transferred onto nitrocellulose membranes. The membranes were incubated with caspase-1 or pro-IL-1β specific antibodies, and then revealed with HRP-conjugated antibody and the ECL system from Amersham (Bucks, UK).

### Crosslinking ASC in PBMCs from malaria patients

PBMCs were resuspended in a hypotonic solution (10 mM Hepes - pH 7.9, 1.5 mM MgCl2, 10 mM KCl, 0.2 mM PMSF, 0.5 mM DTT, protease inhibitor cocktail Roche), incubated on ice for 15 minutes, homogenized (Kontes 22 mm) and centrifuged for 8 minutes at 10,000 *g*. The pellets were resuspended in 500 µl of CHAPs buffer (20 mM HEPES-KOH - pH 7.5, 5 mM MgCl_2_, 0.5 mM EGTA, 0.1% CHAPs, 0.1 mM PMSF, and protease inhibitor cocktail from Roche) and centrifuged for 8 minutes at 10,000 *g*. Finally, the pellets were resuspended in 200 µl of CHAPs buffer, 4 µl of a 100 mM DSS stock solution to a final concentration of 2 mM, and incubated for 30 min in the dark. The oligomers were resolved on a 12% SDS-PAGE and visualized by immunoblotting with an anti-ASC antibody (SC-22514-R).

### Confocal analysis

Cells were fixed with paraformaldehyde 4%, permeabilized using Triton X-100 and stained with anti-NLRP3 (FITC), anti-NLRP12 (Texas Red), NLRC4 (Texas Red) and anti-AIM2 (Texas Red). Images were acquired using a Zeiss LSM510 Microscope and analyzed by ImageJ software. Dual color images were acquired by consecutive scanning with only one laser line active per scan to avoid cross-excitation.

### Cytokine measurements

Measurements of mouse cytokines were performed using commercially available ELISA Duoset kits from R&D Systems. The ranges of detection are 15.6–1000 pg/ml for IL-1β; and 31.2–2000 pg/ml for TNF-α and IFN-γ. Human IL-1β was detected by ELISA kit from Ebioscience in a range of 4 to 500 pg/ml.

### Microarray experiments and data analysis

For mouse experiments we used splenocytes from 3 C57BL/6 and 3 MyD88^−/−^ mice at 6 days p.i. with *P. chabaudi* or uninfected. Gene expression was accessed by microarray analysis using a gene chip from Affymetrix (∼23,000 transcripts). Genes were clustered by Tiger Multi Experiment Viewer software using the fold increase value obtained by the reason of the signal intensity values from infected vs. non-infected mice. Differences in gene expression between the 2 conditions were considered significant if *p*<0.05 as defined by unpaired t test. Detailed methodological and analysis for human microarrays are presented in **Table S1**. Expression Omnibus (http://www.ncbi.nlm.nih.gov/gds/) accession numbers are GSE35083 for mouse and GSE15221 for human microarrays.

### Statistical analysis

All data were analyzed using Graphpad Prism 5.0 Software. Cytokine measurements from human PBMCs were analyzed using two-tailed student's *t* test. Mann-Whitney testing was used for non-parametric analysis when data did not fit a Gaussian distribution. A *p* value≤0.05 was considered to be statistically significant.

## Supporting Information

Figure S1 **LPS challenge in** ***P. chabaudi*** **infected mice is required for expression of pro-IL-1β, but not caspase-1 activation.** C57BL/6 mice were injected with 10^5^ parasitized red blood cells from *P. chabaudi* infected mice. (**A**) Mice at 7 days post-infection (p.i.) were challenged with 10 µg of LPS and spleens harvested 2 hours later. Splenocytes were then labeled with FLICA reagent to assess caspase-1 activation (bottom panels). As a control we used non-labeled splenocytes from infected mice (top panels), and labeled cells from uninfected controls (middle panels). (**B**) Western blot for caspase-1 was performed with lysates of splenocytes from mice at 7 post-infection (left lane). Splenocytes from uninfected mice were cultured in absence of stimuli (right lane) or for 3 hours with LPS (1 µg/ml) and additional 50 min with nigericin (10 µM) (middle lane) as positive control. (**C**) Uninfected control and infected mice were challenged with 10 µg of LPS and spleens harvested 2 hours later. Splenocytes were then lysed and analyzed for active caspase-1 and pro-IL-1β by Western blot. Each group consisted of 4 mice and the results shown are a representative of one out of 2 experiments. A faint band of similar molecular weight of active caspase-1 that corresponds to IgG light chain is seen in the uninfected controls. (**D**) Splenocytes from uninfected and infected mice (at 7 p.i.) were stained with combinations of the following mAbs: T lymphocytes (CD4^+^ or CD8^+^), B lymphocytes (B220^+^) or NK cells (NKG2d^+^), which were shown to be negative for active caspase-1. To each sample, FLICA reagent was added in order to detect active caspase-1. All flow cytometry data were acquired using a LSRII cytometer, DIVA software (BD Biosciences) and analyzed using Flowjo software (TreeStar).
(TIF)

Figure S2 **Parasitemia, cytokine levels and lethality in** ***P. chabaudi*** **infected mice challenged with a low dose of LPS.** (**A**) C57BL/6, ASC^−/−^, and Casp-1^−/−^ mice were injected with 10^5^ parasitized red blood cells. These mice were followed every 3 days for parasitemia evaluation by giemsa stained smears. No significant differences in terms of parasitemia and clinical symptoms were observed when comparing C57BL/6 and the knockout mice. No lethality is observed up to 90 days post-infection. (**B**) C57BL/6 mice were injected with 10^5^ parasitized red blood cells obtained from a *P. chabaudi* infected mouse. Three different groups of mice, (i) uninfected mice (open circles), (ii) at 7 days post-infection (black circles, left panels), and (iii) at 28 days post-infection (black symbols, right panels) were challenged with 10 µg of LPS (intravenously). Sera of five mice from each group (7^th^ and 28^th^ of infection) were harvested 3, 6, and 9 hours post-LPS challenge. Sera from non-challenged mice were also collected and used as time zero. The levels of cytokines were quantified by an ELISA assay. Lethality was evaluated at 48 hours post-LPS challenge, in another 2 groups of mice that have been infected for 7 or 28 days.
(TIF)

Figure S3 **Role of TLR9 in** ***Plasmodium*** **-induced inflammatory priming.** C57BL6 and TLR9^−/−^ mice were infected with 10^5^ parasitized red blood cells. (**A**) At 7 days of *P. chabaudi* infection spleens were harvested, and the splenocyte lysates used to detect active caspase-1 by Western blot assay. (**B**) Mice were challenged with 10 µg of LPS, sera collected 8 hours later, and levels of IL-1β quantified by ELISA. Five mice were used per group and statistical analysis performed by Student's t-test indicate that differences are statistically significant (** *p = 0.0032* and *** *p<0.0001*). (**C**) Mice were challenged with 10 µg of LPS and survival evaluated for 48 hours. Fisher's exact test was used to analyze the results and significant differences indicated by an asterisk (*p = 0.0245*). (**D - top panel**) At 7 days post-infection C57BL/6 mice were challenged with 10 µg of LPS or 200 µg of CpG ODN 7909 and lethality evaluated for 72 hours. (**D - bottom panel**) Non-infected C57BL/6 mice were primed with 100 µg of CpG ODN 7909 or 250 µg of sHz. Six hours later mice were treated or not with IL-1R antagonist (100 mg/kg) and challenged with 50 µg of LPS. For the results shown in **Panel D** five mice were used per group. The results are representative of 2 experiments that yield similar results. The statistic analysis was performed employing the Fisher's exact test and differences indicated by an asterisk (*p = 0.0132*).
(TIF)

Figure S4 **Requirement of endogenous IFN-γ and functional TNFR1 for caspase-1 activation and pro-IL-1β expression.** C57BL6, IFN-γ^−/−^ and TNFR1^−/−^ mice were infected with 10^5^ parasitized red blood cells. (**A**) At 7 days post-infection spleens were harvested and splenocyte lysates used in a Western Blot to detect active caspase-1. (**B**) At 7 days post–infection mice were challenged with 10 µg of LPS. Two hours later spleens were harvested and cell lysates used to detect pro-IL-1β in a Western blot.
(TIF)

Figure S5 **Caspase-1 expression and activation in malaria or IFN-γ primed monocytes.** PBMCs from acutely *P. vivax* infected patients were stained with combinations of the mAbs specific for: (**A**) Histograms were performed based on CD14^+^CD16^−^ gated monocytes (left column) and on CD14^dim^CD16^+^ gated monocytes (right column). Active caspase-1 was evaluated by FLICA reagent (top panel), membrane integrity by nuclei staining with 7AAD (middle panel), and cell size change by shift on FSC axis (bottom panel). (**B**) Gate strategy and histograms are also shown for T lymphocytes (CD3^+^/CD4^+^ or CD3^+^/CD8^+^), B Cells (CD19^+^), myeloid dendritic cells (CD1c^+^/CD19^−^) and plasmacytoid dendritic cells (CD123^+^/CD303^+^). To each sample, FLICA reagent and 7AAD were added as indicated, and analyzed for caspase-1 activity, membrane damage and cell size (FSC). The data were acquired using a LSRII cytometer, DIVA software (BD Biosciences) and analyzed using Flowjo software (TreeStar). (**C**) Western blot analysis reveal active caspase-1 (p10) in lysates from PBMCs of six *P. vivax* infected patients, but not from the healthy controls. (**D**) Microarray analysis was performed in PBMCs from 14 *P. falciparum* malaria patients during malaria sepsis and 30–40 days post-treatment and parasitological cure. The presented data were calculated by establishing the fold increase on gene expression, when comparing the same patient before and after treatment. (**E**) Monocytes from healthy donors were stimulated with either or both LPS (100 ng/ml) and IFN-γ for 24 hours, and the levels of pro-caspase-1 and pro-IL-1β detected in the cell lysate by Western blot. Pro-caspase-1 and Pro-IL-1β expression was quantified by densitometric analysis. The levels of IL-1β produced by stimulated monocytes were determined in the cell culture supernatants by ELISA. Significant differences are **p<0.005 and ***p<*0.001* as indicated by the unpaired *t* test with Welch correction or Mann-Whitney test when a normality test failed.
(TIF)

Figure S6 **Transfected HEK cells expressing cytosolic receptors and LPS + ATP-induced NLRP3 specks.** (**Top**) Confocal analysis detected NLRP3 specks (green) in monocytes activated with LPS and nigericin, as well as diffuse NLRP12, NLRC4 and AIM2 in cells transfected with the respective plasmid. (**Bottom**) Western blots of THP-1 cells, as well as HEK cells (negative controls) and HEK cells transfected with plasmids encoding NLRP12, NLRC4 and AIM2. Reaction with secondary antibodies in the absence of primary antibody or non-transfected HEK cells yielded negative results both on western blots or confocal analysis.
(TIF)

Table S1 **DNA microarray during murine malaria.** The expression value for each gene was determined by calculating the average of differences in intensity (perfect match intensity – mismatch intensity) between its probe pairs. The expression analysis file created from each sample (chip) was imported into GeneSpring 7.2 (Agilent Technologies, Inc., Palo Alto, CA) for further data characterization. A new experiment was generated after importing data from the same organ in which data were normalized by the array to the 50% percentile of all measurements on that array. Lists of genes that were either induced or suppressed between samples from non-infected vs. 6 days infected mice of same genotype were created by filtration-on-fold function within the presented list. The differential expression was evaluated in grouped samples from each mouse genotype (WT or knockout mice) and time point (non-infected vs 6 days post-infection) using ANOVA.
(PPT)

Table S2 **DNA microarray during human malaria.** This microarray involved 1 4 patients that were analyzed employing the Illumina HumanWG-6 v2.0 (∼47,000 transcripts microchip (Franklin et al., 2009). The malaria patients from Porto Velho, Brazil, were naturally infected with *P. falciparum* and analyzed before and after documented curative treatment with mefloquine. PCR was used for excluding patients with mixed infections involving *P. vivax*. Each patient served as his/her own control. Fold increase of gene expression was calculated by using PBMC of the same patient before and 30–40 days after therapy. Sample filtering, normalization and averaging were carried out with BASE (Bio-Array Software Environment) and log transformed (log2). Genes were filtered to include only genes with a signal greater than the average signal from the negative controls in at least one of the samples with a detection *p* value less than 0.01. Differences in gene expression between the two conditions were considered significant if *p*<0.01 with a paired t test with Benjamini-Hochberg correction for multiple testing and a fold change equal or greater than 1.7.
(PPT)
