# Correction: Malaria-Induced NLRP12/NLRP3-Dependent Caspase-1 Activation Mediates Inflammation and Hypersensitivity to Bacterial Superinfection

**DOI:** 10.1371/journal.ppat.1004258

**Published:** 2014-06-20

**Authors:** The PLOS Pathogens Staff

There is a misplacement of loading controls (β-actin) in the original Figures 2D and S4A (left panel). The corrected version of Figure 2D and Figure S4A can be seen here. Since these changes had no implications in any of the results and conclusions of the study, there is no further change in Figure legends or main text of the manuscript.

**Figure 2:**
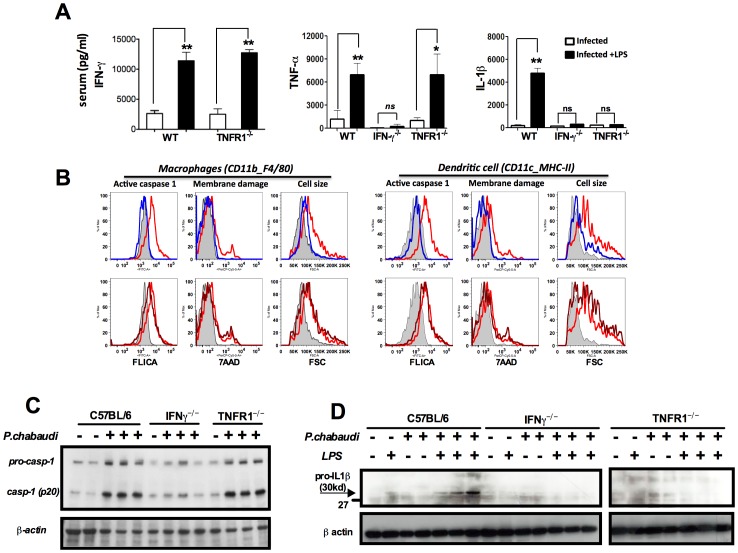
Both endogenous IFN-γ and TNF-α are required for IL-1β production in mice infected with *P. chabaudi*. (**A**) Mice were challenged with 10 µg of LPS, at 7 days post-infection, and sera collected 9 hours later for cytokine measurements. These results are means + SEM of 10 animals from 2 independent experiments. Significant differences are *p  =  0.034 and **p  =  0.001 as indicated by the Mann-Whitney test. (**B**) Active caspase-1, membrane integrity and cell size were assessed in macrophages (CD11b^+^F4/80^+^) and DCs (CD11c^+^MHC-II^+^) by flow cytometry, employing the FLICA reagent, nuclei staining with 7AAD, and the shift on FSC axis, respectively. These results presented in figures are representative of 2 experiments. (**C**) Splenocytes lysates were obtained from mice at 7 days post-infection and used in Western blot analysis. A faint band of similar molecular weight of active caspase-1 that corresponds to IgG light chain is seen in the uninfected controls or infected IFN-γ^−/−^ mice. These results presented in figures are representative of 2 experiments. (**D**) Two hours after LPS-challenge, splenocytes lysates were harvested to evaluate expression of pro-IL-1β. These results are representative of 3 independent experiment that yielded similar results.

## Supporting Information

Figure S4 **Requirement of endogenous IFN-γ and functional TNFR1 for caspase-1 activation and pro-IL-1β expression.** C57BL6, IFN-γ^−/−^ and TNFR1^−/−^ mice were infected with 10^5^ parasitized red blood cells. (A) At 7 days post-infection spleens were harvested and splenocyte lysates used in a Western Blot to detect active caspase-1. (B) At 7 days post–infection mice were challenged with 10 µg of LPS. Two hours later spleens were harvested and cell lysates used to detect pro-IL-1β in a Western blot.
(TIF)
