# Supplementary figures and images for: Malaria-Induced NLRP12/NLRP3-Dependent Caspase-1 Activation Mediates Inflammation and Hypersensitivity to Bacterial Superinfection
Source: PLoS Pathog. 2014 Jan 16;10(1):e1003885. doi: 10.1371/journal.ppat.1003885 (PMC3894209; doi:10.1371/journal.ppat.1003885)

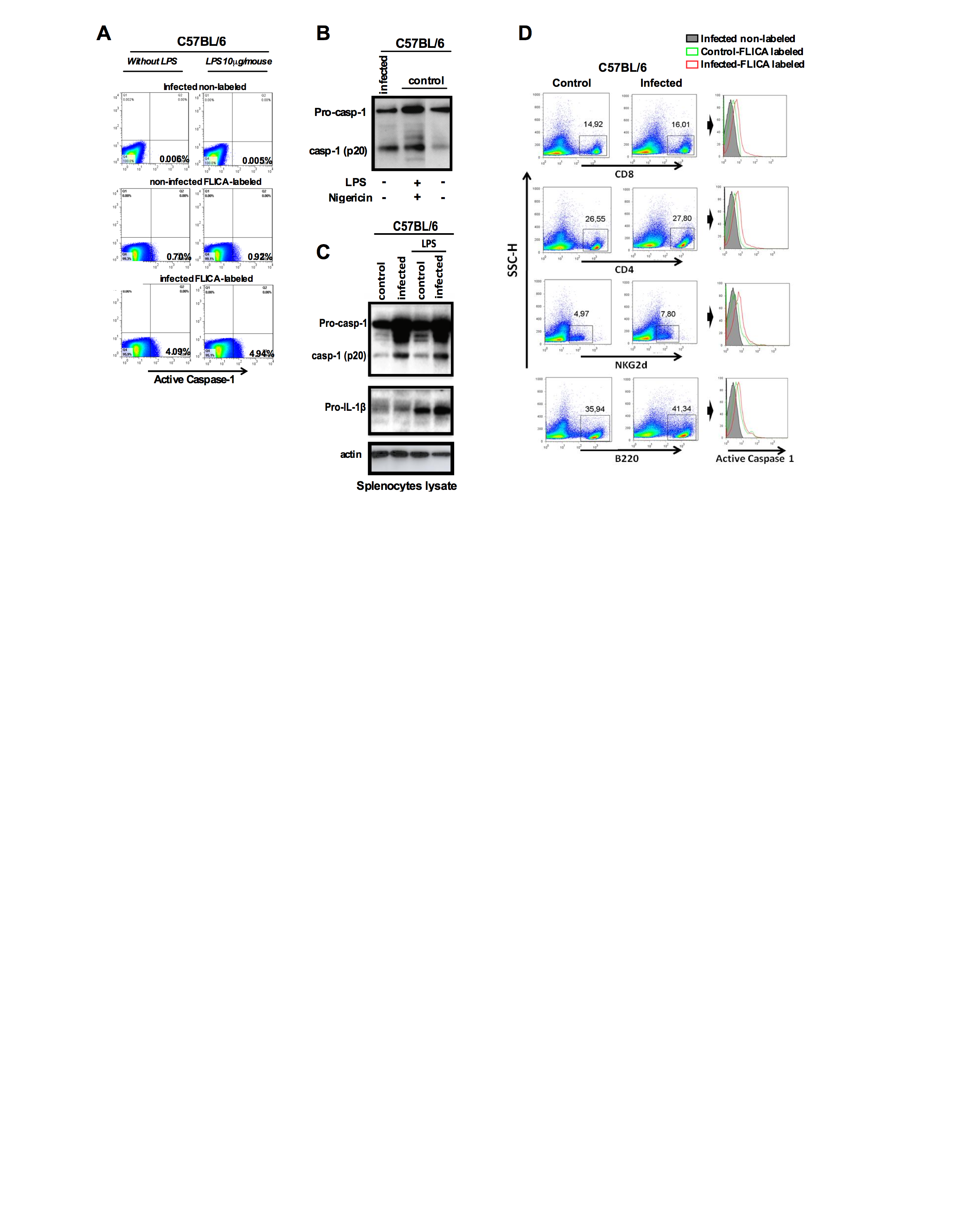

Supplement: Figure S1 — LPS challenge in P. chabaudi infected mice is required for expression of pro-IL-1β, but not caspase-1 activation. C57BL/6 mice were injected with 105 parasitized red blood cells from P. chabaudi infected mice. (A) Mice at 7 days post-infection (p.i.) were challenged with 10 µg of LPS and spleens harvested 2 hours later. Splenocytes were then labeled with FLICA reagent to assess caspase-1 activation (bottom panels). As a control we used non-labeled splenocytes from infected mice (top panels), and labeled cells from uninfected controls (middle panels). (B) Western blot for caspase-1 was performed with lysates of splenocytes from mice at 7 post-infection (left lane). Splenocytes from uninfected mice were cultured in absence of stimuli (right lane) or for 3 hours with LPS (1 µg/ml) and additional 50 min with nigericin (10 µM) (middle lane) as positive control. (C) Uninfected control and infected mice were challenged with 10 µg of LPS and spleens harvested 2 hours later. Splenocytes were then lysed and analyzed for active caspase-1 and pro-IL-1β by Western blot. Each group consisted of 4 mice and the results shown are a representative of one out of 2 experiments. A faint band of similar molecular weight of active caspase-1 that corresponds to IgG light chain is seen in the uninfected controls. (D) Splenocytes from uninfected and infected mice (at 7 p.i.) were stained with combinations of the following mAbs: T lymphocytes (CD4+ or CD8+), B lymphocytes (B220+) or NK cells (NKG2d+), which were shown to be negative for active caspase-1. To each sample, FLICA reagent was added in order to detect active caspase-1. All flow cytometry data were acquired using a LSRII cytometer, DIVA software (BD Biosciences) and analyzed using Flowjo software (TreeStar). (TIF) [file ppat.1003885.s001.tif]

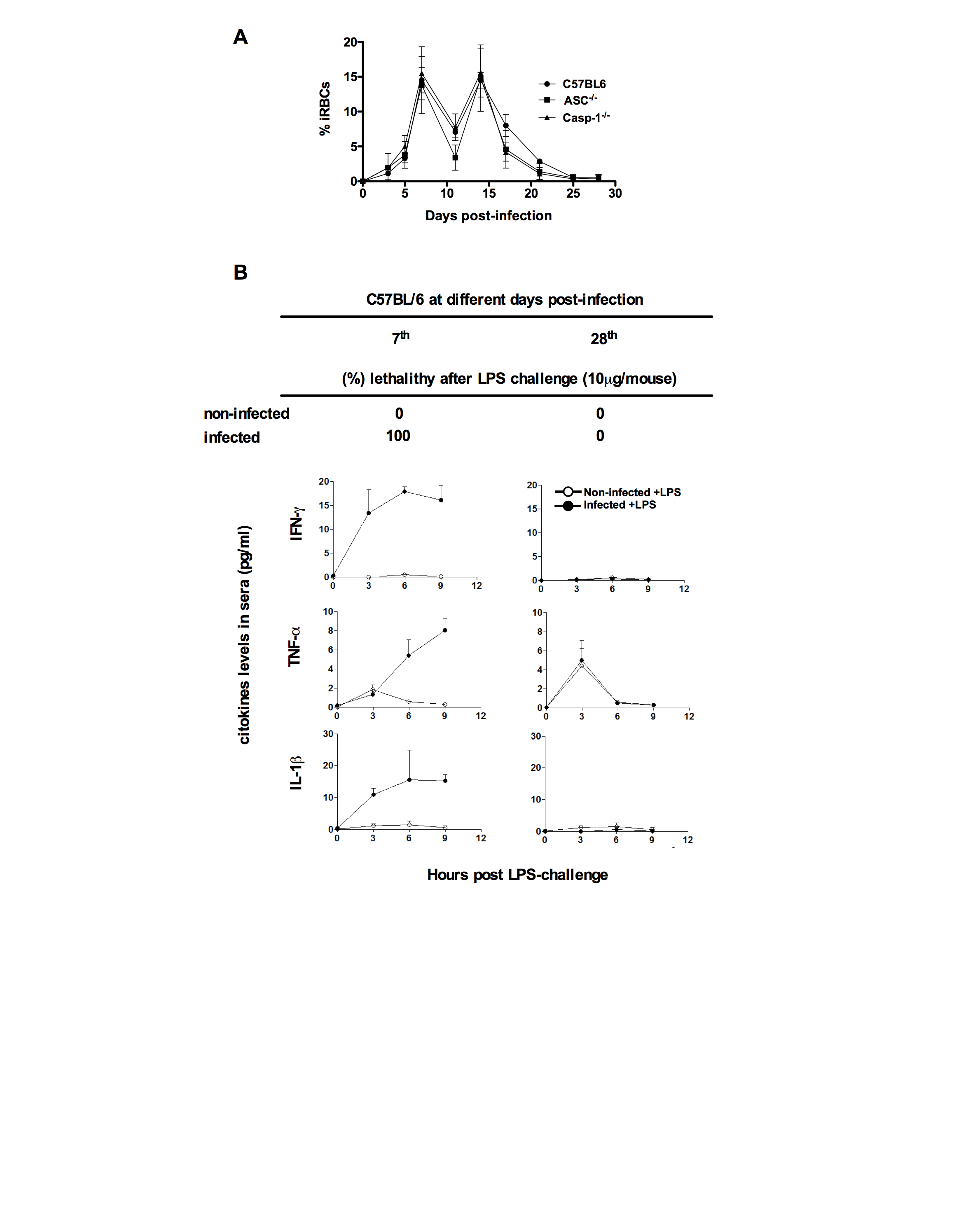

Supplement: Figure S2 — Parasitemia, cytokine levels and lethality in P. chabaudi infected mice challenged with a low dose of LPS. (A) C57BL/6, ASC−/−, and Casp-1−/− mice were injected with 105 parasitized red blood cells. These mice were followed every 3 days for parasitemia evaluation by giemsa stained smears. No significant differences in terms of parasitemia and clinical symptoms were observed when comparing C57BL/6 and the knockout mice. No lethality is observed up to 90 days post-infection. (B) C57BL/6 mice were injected with 105 parasitized red blood cells obtained from a P. chabaudi infected mouse. Three different groups of mice, (i) uninfected mice (open circles), (ii) at 7 days post-infection (black circles, left panels), and (iii) at 28 days post-infection (black symbols, right panels) were challenged with 10 µg of LPS (intravenously). Sera of five mice from each group (7th and 28th of infection) were harvested 3, 6, and 9 hours post-LPS challenge. Sera from non-challenged mice were also collected and used as time zero. The levels of cytokines were quantified by an ELISA assay. Lethality was evaluated at 48 hours post-LPS challenge, in another 2 groups of mice that have been infected for 7 or 28 days. (TIF) [file ppat.1003885.s002.tif]

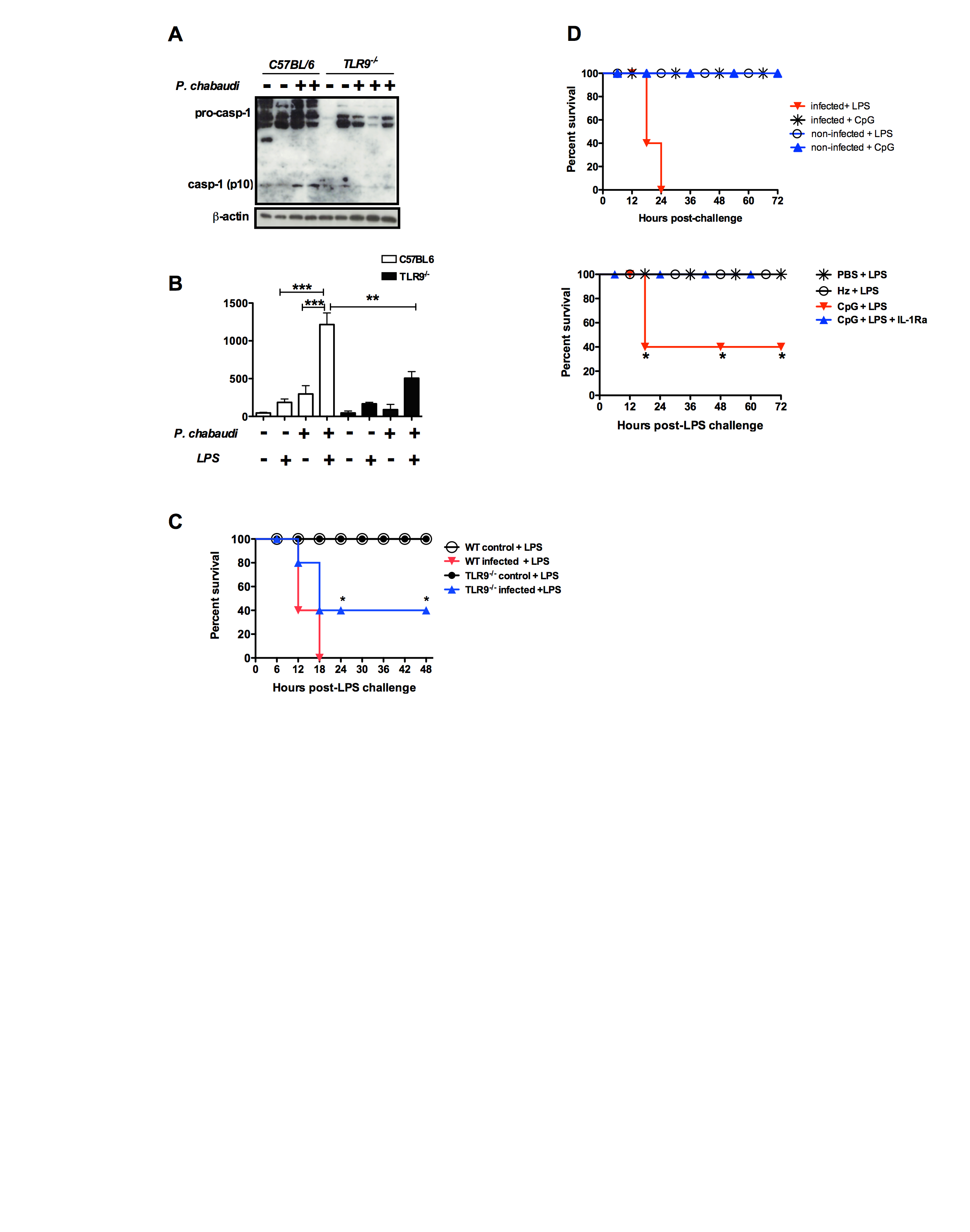

Supplement: Figure S3 — Role of TLR9 in Plasmodium -induced inflammatory priming. C57BL6 and TLR9−/− mice were infected with 105 parasitized red blood cells. (A) At 7 days of P. chabaudi infection spleens were harvested, and the splenocyte lysates used to detect active caspase-1 by Western blot assay. (B) Mice were challenged with 10 µg of LPS, sera collected 8 hours later, and levels of IL-1β quantified by ELISA. Five mice were used per group and statistical analysis performed by Student's t-test indicate that differences are statistically significant (** p = 0.0032 and *** p<0.0001). (C) Mice were challenged with 10 µg of LPS and survival evaluated for 48 hours. Fisher's exact test was used to analyze the results and significant differences indicated by an asterisk (p = 0.0245). (D - top panel) At 7 days post-infection C57BL/6 mice were challenged with 10 µg of LPS or 200 µg of CpG ODN 7909 and lethality evaluated for 72 hours. (D - bottom panel) Non-infected C57BL/6 mice were primed with 100 µg of CpG ODN 7909 or 250 µg of sHz. Six hours later mice were treated or not with IL-1R antagonist (100 mg/kg) and challenged with 50 µg of LPS. For the results shown in Panel D five mice were used per group. The results are representative of 2 experiments that yield similar results. The statistic analysis was performed employing the Fisher's exact test and differences indicated by an asterisk (p = 0.0132). (TIF) [file ppat.1003885.s003.tif]

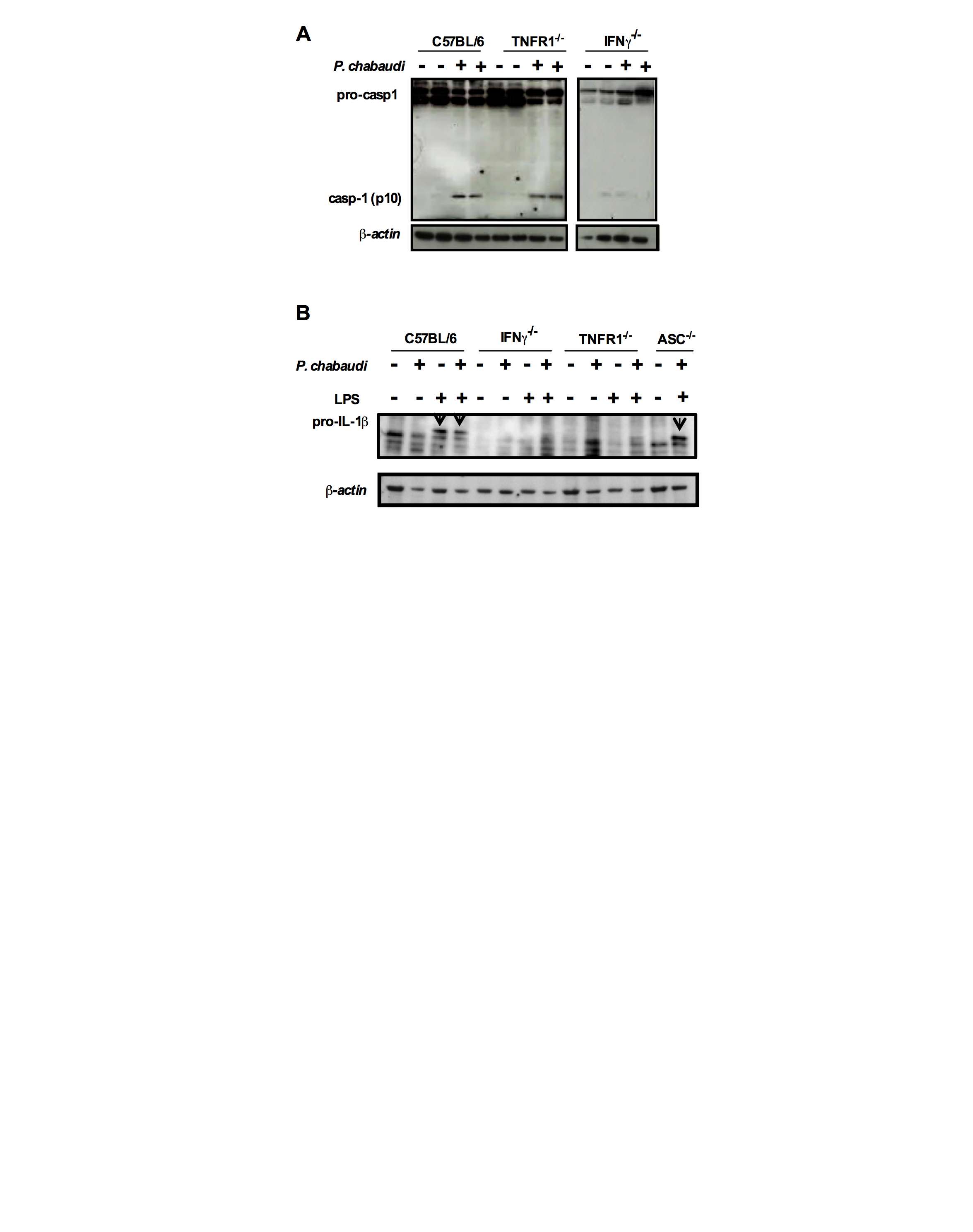

Supplement: Figure S4 — Requirement of endogenous IFN-γ and functional TNFR1 for caspase-1 activation and pro-IL-1β expression. C57BL6, IFN-γ−/− and TNFR1−/− mice were infected with 105 parasitized red blood cells. (A) At 7 days post-infection spleens were harvested and splenocyte lysates used in a Western Blot to detect active caspase-1. (B) At 7 days post–infection mice were challenged with 10 µg of LPS. Two hours later spleens were harvested and cell lysates used to detect pro-IL-1β in a Western blot. (TIF) [file ppat.1003885.s004.tif]

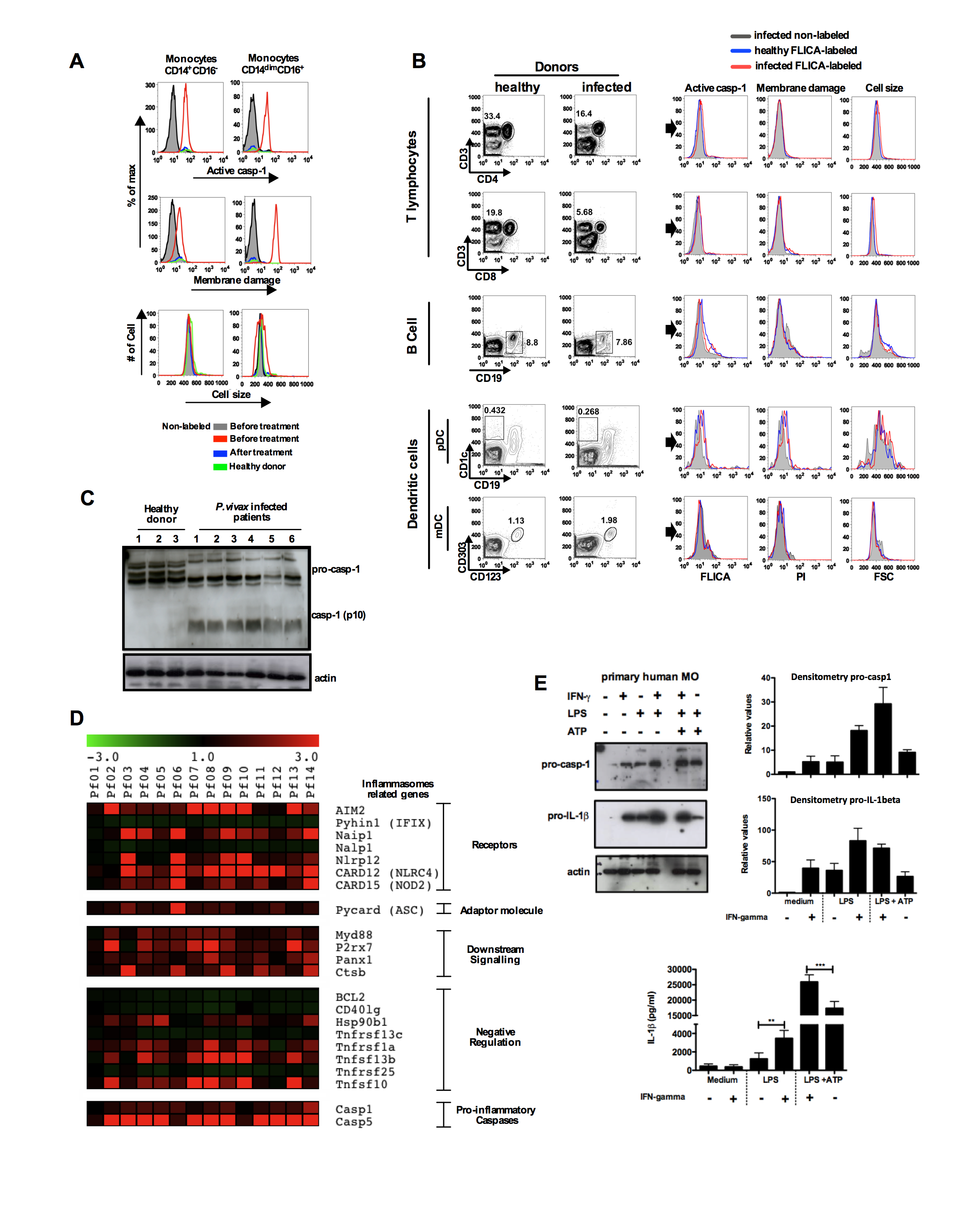

Supplement: Figure S5 — Caspase-1 expression and activation in malaria or IFN-γ primed monocytes. PBMCs from acutely P. vivax infected patients were stained with combinations of the mAbs specific for: (A) Histograms were performed based on CD14+CD16− gated monocytes (left column) and on CD14dimCD16+ gated monocytes (right column). Active caspase-1 was evaluated by FLICA reagent (top panel), membrane integrity by nuclei staining with 7AAD (middle panel), and cell size change by shift on FSC axis (bottom panel). (B) Gate strategy and histograms are also shown for T lymphocytes (CD3+/CD4+ or CD3+/CD8+), B Cells (CD19+), myeloid dendritic cells (CD1c+/CD19−) and plasmacytoid dendritic cells (CD123+/CD303+). To each sample, FLICA reagent and 7AAD were added as indicated, and analyzed for caspase-1 activity, membrane damage and cell size (FSC). The data were acquired using a LSRII cytometer, DIVA software (BD Biosciences) and analyzed using Flowjo software (TreeStar). (C) Western blot analysis reveal active caspase-1 (p10) in lysates from PBMCs of six P. vivax infected patients, but not from the healthy controls. (D) Microarray analysis was performed in PBMCs from 14 P. falciparum malaria patients during malaria sepsis and 30–40 days post-treatment and parasitological cure. The presented data were calculated by establishing the fold increase on gene expression, when comparing the same patient before and after treatment. (E) Monocytes from healthy donors were stimulated with either or both LPS (100 ng/ml) and IFN-γ for 24 hours, and the levels of pro-caspase-1 and pro-IL-1β detected in the cell lysate by Western blot. Pro-caspase-1 and Pro-IL-1β expression was quantified by densitometric analysis. The levels of IL-1β produced by stimulated monocytes were determined in the cell culture supernatants by ELISA. Significant differences are **p<0.005 and ***p<0.001 as indicated by the unpaired t test with Welch correction or Mann-Whitney test when a normality test failed. (TIF) [file ppat.1003885.s005.tif]

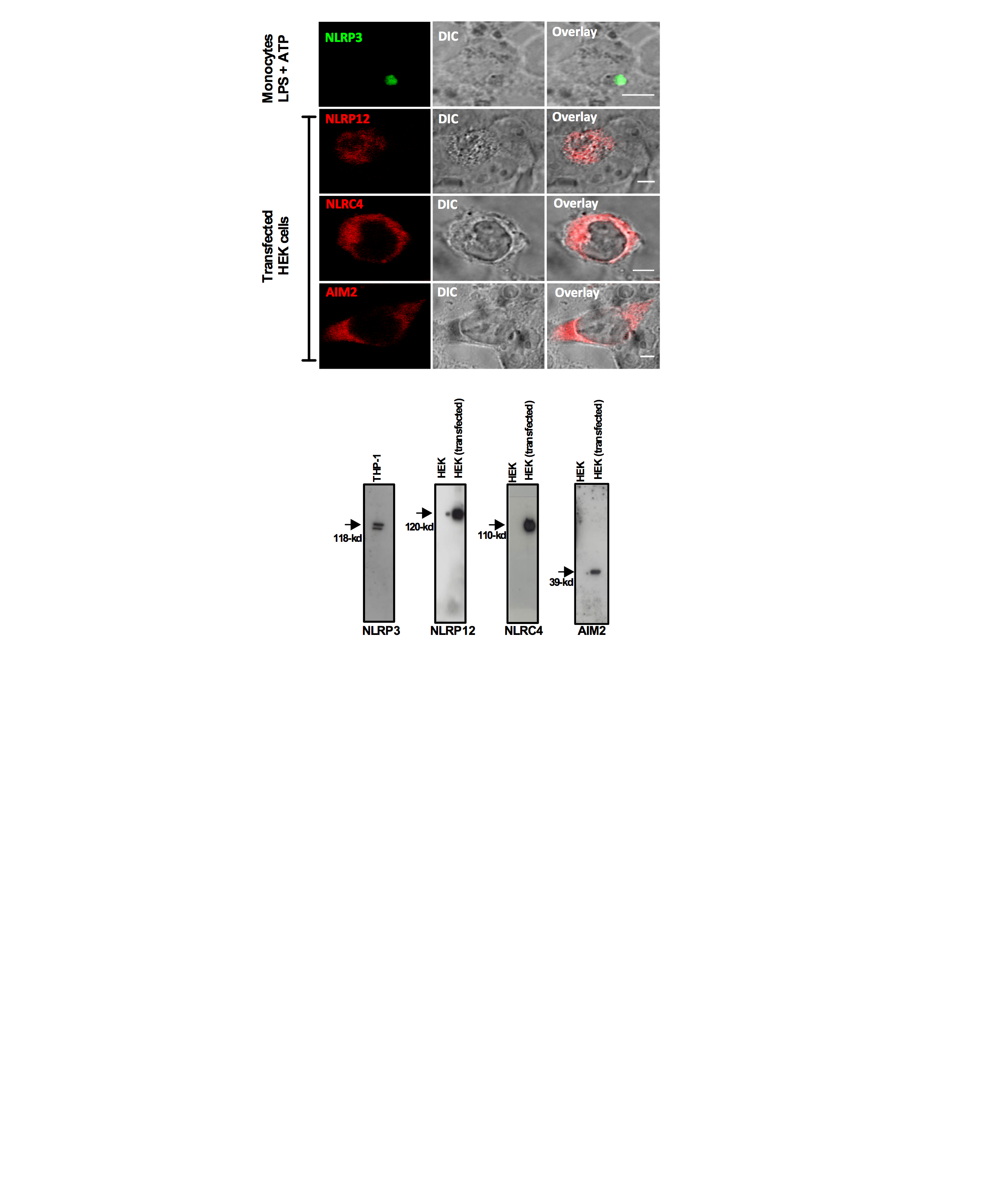

Supplement: Figure S6 — Transfected HEK cells expressing cytosolic receptors and LPS + ATP-induced NLRP3 specks. (Top) Confocal analysis detected NLRP3 specks (green) in monocytes activated with LPS and nigericin, as well as diffuse NLRP12, NLRC4 and AIM2 in cells transfected with the respective plasmid. (Bottom) Western blots of THP-1 cells, as well as HEK cells (negative controls) and HEK cells transfected with plasmids encoding NLRP12, NLRC4 and AIM2. Reaction with secondary antibodies in the absence of primary antibody or non-transfected HEK cells yielded negative results both on western blots or confocal analysis. (TIF) [file ppat.1003885.s006.tif]
