# Supplementary figures and images for: Correction: Malaria-Induced NLRP12/NLRP3-Dependent Caspase-1 Activation Mediates Inflammation and Hypersensitivity to Bacterial Superinfection
Source: PLoS Pathog. 2014 Jun 20;10(6):e1004258. doi: 10.1371/journal.ppat.1004258 (PMC4065140; doi:10.1371/journal.ppat.1004258)

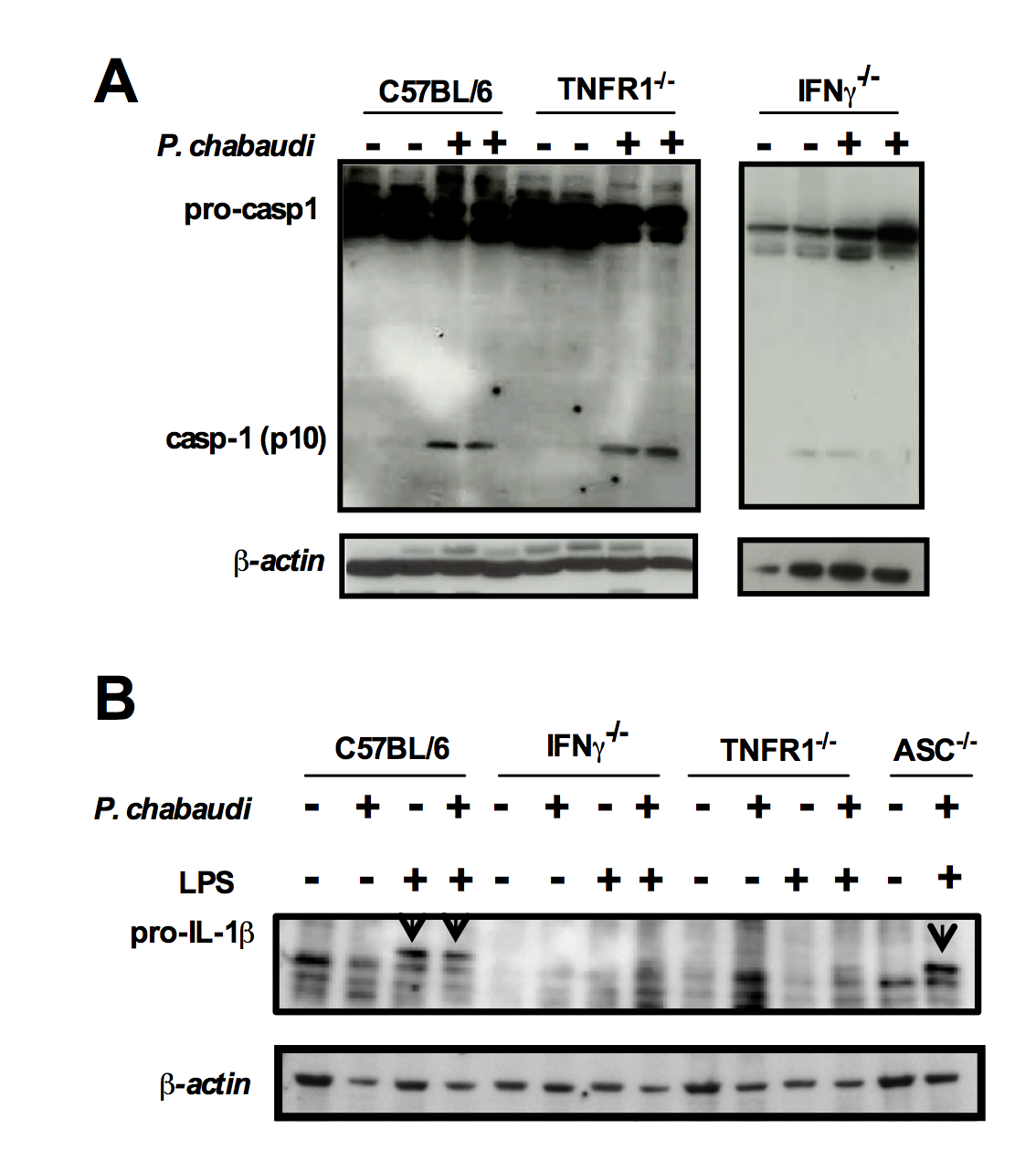

Supplement: Figure S4 — Requirement of endogenous IFN-γ and functional TNFR1 for caspase-1 activation and pro-IL-1β expression. C57BL6, IFN-γ−/− and TNFR1−/− mice were infected with 105 parasitized red blood cells. (A) At 7 days post-infection spleens were harvested and splenocyte lysates used in a Western Blot to detect active caspase-1. (B) At 7 days post–infection mice were challenged with 10 µg of LPS. Two hours later spleens were harvested and cell lysates used to detect pro-IL-1β in a Western blot. (TIF) [file ppat.1004258.s001.tif]
